# Time-Dependent Rate Phenomenon in Viruses

**DOI:** 10.1128/JVI.00593-16

**Published:** 2016-07-27

**Authors:** Pakorn Aiewsakun, Aris Katzourakis

**Affiliations:** Department of Zoology, University of Oxford, Oxford, United Kingdom; University of Illinois at Chicago

## Abstract

Among the most fundamental questions in viral evolutionary biology are how fast viruses evolve and how evolutionary rates differ among viruses and fluctuate through time. Traditionally, viruses are loosely classed into two groups: slow-evolving DNA viruses and fast-evolving RNA viruses. As viral evolutionary rate estimates become more available, it appears that the rates are negatively correlated with the measurement timescales and that the boundary between the rates of DNA and RNA viruses might not be as clear as previously thought. In this study, we collected 396 viral evolutionary rate estimates across almost all viral genome types and replication strategies, and we examined their rate dynamics. We showed that the time-dependent rate phenomenon exists across multiple levels of viral taxonomy, from the Baltimore classification viral groups to genera. We also showed that, by taking the rate decay dynamics into account, a clear division between the rates of DNA and RNA viruses as well as reverse-transcribing viruses could be recovered. Surprisingly, despite large differences in their biology, our analyses suggested that the rate decay speed is independent of viral types and thus might be useful for better estimation of the evolutionary time scale of any virus. To illustrate this, we used our model to reestimate the evolutionary timescales of extant lentiviruses, which were previously suggested to be very young by standard phylogenetic analyses. Our analyses suggested that these viruses are millions of years old, in agreement with paleovirological evidence, and therefore, for the first time, reconciled molecular analyses of ancient and extant viruses.

**IMPORTANCE** This work provides direct evidence that viral evolutionary rate estimates decay with their measurement timescales and that the rate decay speeds do not differ significantly among viruses despite the vast differences in their molecular features. After adjustment for the rate decay dynamics, the division between the rates of double-stranded DNA (dsDNA), single-stranded RNA (ssRNA), and ssDNA/reverse-transcribing viruses could be seen more clearly than before. Our results provide a guideline for further improvement of the molecular clock. As a demonstration of this, we used our model to reestimate the timescales of modern lentiviruses, which were previously thought to be very young, and concluded that they are millions of years old. This result matches the estimate from paleovirological analyses, thus bridging the gap between ancient and extant viral evolutionary studies.

## INTRODUCTION

An accurate and precise knowledge of the rate of viral evolution is central to the reconstruction of viral natural history, which is necessary for the calculation of many evolutionary parameters, from viral age estimates to population size. Generally, viruses are loosely classed into two groups according to their rates of evolution: “slow-evolving” and “fast-evolving” viruses. DNA viruses, especially double-stranded DNA (dsDNA) viruses, are traditionally thought of as slow-evolving viruses. To estimate molecular evolutionary rates, the absolute timescales for the observed genetic differences are required, and these can be derived from the divergence dates and/or sampling dates of the study subjects. Many dsDNA viruses have been shown to have an extremely stable cospeciation history with their hosts, and therefore, their divergence dates can be directly inferred from those of their hosts. On the basis of these observations, their rates have been estimated to be of the order of 10^−7^ to 10^−9^ nucleotide substitutions per site per year (s/n/y) (1–5), comparable to those of their hosts (6, 7).

RNA viruses, on the other hand, are typically regarded as fast-evolving viruses. RNA viruses are generally characterized by frequent cross-species transmissions in nature; as a result, it is often difficult to calibrate their evolutionary rates using host evolutionary timescales. Their rates are thus often calculated by using molecular sequences collected at different time points (heterochronous molecular data sets). In this case, the differences among sampling times provide the timescales for the observed genetic divergence. Based on these analyses, their rates are commonly estimated to be between 10^−2^ and 10^−5^ s/n/y (8–11), 2 to 7 orders of magnitude higher than the typical rates of dsDNA viruses.

This conventional concept of a dichotomy between the rates of DNA and RNA viral evolution has recently been challenged, however, and it seems that the boundary between the rates of DNA and RNA viruses might not be as clear as previously thought (11–14). For example, analyses of heterochronous molecular data sets of dsDNA and single-stranded DNA (ssDNA) viruses has revealed that DNA viruses are in fact capable of evolving very rapidly over short timescales, with rates ranging between 10^−3^ and 10^−6^ s/n/y (15–19), comparable to the established rates of many RNA viruses. On the other hand, when the cospeciation assumption is applicable to RNA viruses, such as deltaretroviruses, hantaviruses, and foamy viruses, analyses suggest that their long-term rates of evolution are extremely low, estimated to be in the range of 10^−7^ to 10^−8^ s/n/y (20–23), comparable to those of dsDNA viruses. Paleovirological analyses have also shown that many ancient endogenous viruses related to RNA viruses exhibit high similarity to their modern-day counterparts despite being millions of years old (24). This provides independent evidence indicating that RNA viruses can indeed evolve very slowly over geological timescales (24). If one naïvely combines all the rate estimates, the ranges of the rate estimates for both DNA and RNA viral evolution would appear to be extremely wide, spanning 10^−3^ to 10^−9^ s/n/y for DNA viruses and 10^−2^ to 10^−8^ s/n/y for RNA viruses, largely overlapping with one another. As a result, there is an emerging consensus that there is no strict division between the evolutionary rates of DNA and RNA viruses (13, 14).

As viral evolutionary rate estimates become more available, it is becoming increasingly clear that viral evolutionary rates appear to vary over time, continuously decreasing with the timescale of rate measurement (25–27). Many hypotheses have been proposed to explain this time-dependent rate phenomenon (TDRP), including temporal changes in selection pressure and/or viral biology, as well as the facts that short-term rates are methodologically prone to overestimation and long-term rates tend to be underestimated (for a review, see reference 25). This phenomenon may at least partly explain the observed large variation and overlap of viral evolutionary rate estimates.

Another consequence of the TDRP is that naïvely transferring the rate estimates over different timescales for evolutionary inference can severely bias the outcome (25–27). The best illustration of this is perhaps the severe underestimation of the evolutionary timescales of extant viruses by current standard phylogenetic tools. For example, while paleovirological analyses unequivocally show that simian immunodeficiency viruses (SIVs) are millions of years old (28, 29), all previous standard phylogenetic analyses, which do not account for the TDRP, have suggested that they are young, with the most recent common ancestor (MRCA) dating to less than a million years ago (8, 30–32). One pragmatic approach to solving this problem is to use models describing the empirical relationship between the rate estimates and their measurement timescales to correct for the TDRP effects in evolutionary analyses (27, 33). Our study of foamy viruses has shown that a power law rate decay model can describe the TDRP very well empirically, and thus, it may be useful as a tool for correcting for the effects of the TDRP (27).

In this study, we collected 396 viral nucleotide substitution rates across almost all viral molecular features and replication strategies, and we examined their TDRP dynamics at various taxonomical levels by using the power law rate decay model as the basis of our investigation. We also examined how the rate dynamics differ among viruses and reexamined the concept of fast- and slow-evolving viruses. Last, we demonstrated the use of our TDRP model by estimating the evolutionary timescale of extant lentiviruses, which has always been severely underestimated by standard phylogenetic analyses.

## MATERIALS AND METHODS

### Data collection and construction of phylogenetically independent data sets of rate estimates.

**FIG 1:**
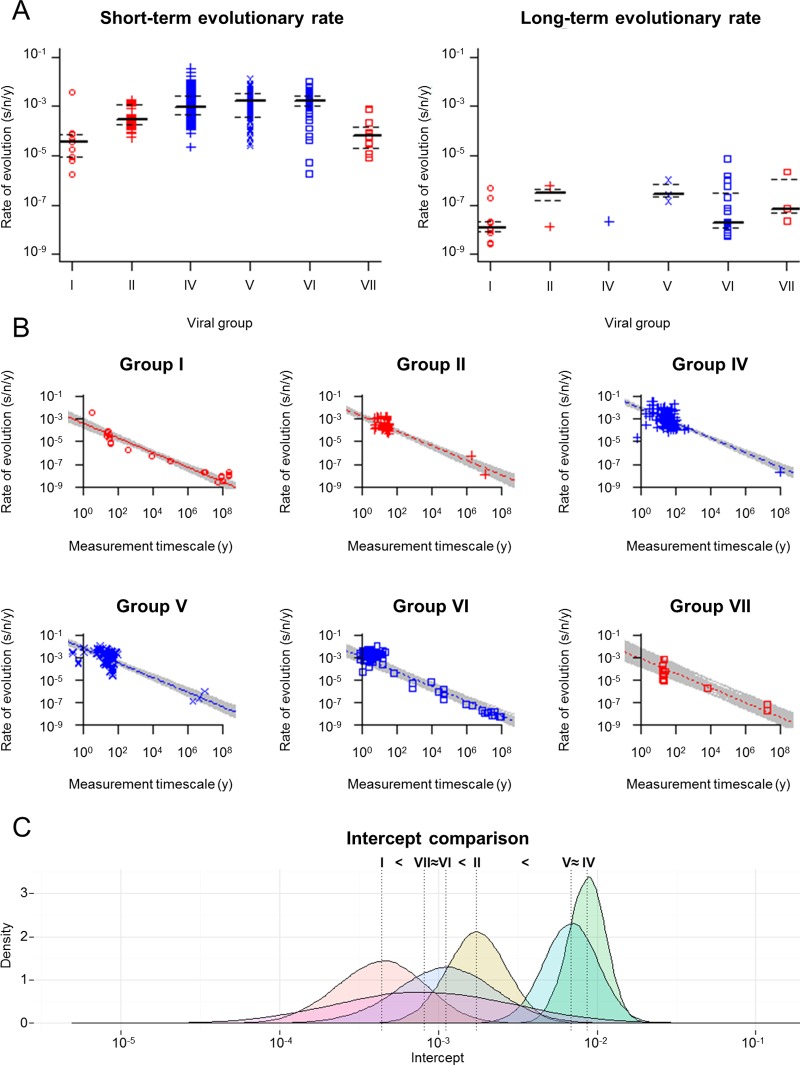
Time-dependent rate phenomenon among viral groups. (A) A total of 359 viral short-term rate estimates (calculated over timescales of 0.16 to 760 years) (left), and 37 long-term rate estimates (calculated over timescales of 6,600 to 2.28 × 10^8^ years) (right) were collected from 133 publications, including 21 rate estimates for group I dsDNA viruses, 47 for group II ssDNA viruses, 123 for group IV (+)ssRNA viruses, 106 for group V (−)ssRNA viruses, 85 for group VI RT-RNA viruses, and 14 for group VII RT-DNA viruses (Table S1 in the supplemental material). Lines indicating the upper quartile (top dashed line), median (central solid line), and lower quartile (bottom dashed line) were added to each viral group to aid visualizing the point's density. (B) Viral evolutionary rate estimates are negatively correlated with their measurement timescales. Gray lines represent 1,000 individual best-fit models, where the slopes are the same across all viral groups. Red or blue lines represent models that are parameterized by median parameter estimates; open red circles and solid line, group I dsDNA viruses; red plus signs and dashed line, group II ssDNA viruses; blue plus signs and dashed line, group IV (+)ssRNA viruses; blue crosses and long-dashed line, group V (−)ssRNA viruses; open blue squares and dotted line, group VI RT-RNA viruses; open red squares and dotted line, group VII RT-DNA viruses. (C) Complete pairwise comparisons of the intercepts of the rate decay curves (representing rate estimates controlled for a 1-year timescale of rate measurement). Vertical dotted lines indicate median estimates. The differences among intercepts were evaluated at a significance level of 0.05.

A total of 396 viral nucleotide substitution rate estimates were collected from 133 pieces of published literature. The rates were arbitrarily divided into two groups: short-term rates (estimated over timescales of <1,000 years) and long-term rates (estimated over timescales of >1,000 years) (Fig. 1A; see also Table S1 in the supplemental material). We considered 1,000 years to be an appropriate cutoff because it corresponds to a large gap in the rate measurement timescales (between 760 years and 6,600 years), clearly dividing rate estimates into two distinct rate categories. To control for the quality of the data, only evolutionary rates estimated under the Bayesian or maximum likelihood frameworks or from neighbor-joining trees were collected. All the rates estimated from neighbor-joining trees were calculated based on explicit nucleotide substitution models where the rates of transversion and transition substitutions were distinguished, at the very least. In two studies however, the models were not reported. In total, the data set comprises nucleotide substitution rate estimates of six major viral groups (according to the Baltimore classification), including 21 rate estimates for group I dsDNA viruses, 47 for group II ssDNA viruses, 123 for group IV positive ssRNA [(+)ssRNA] viruses, 106 for group V negative ssRNA [(−)ssRNA] viruses, 85 for group VI reverse-transcribing RNA (RT-RNA) viruses and 14 for group VII reverse-transcribing DNA (RT-DNA) viruses (Table 1).

**TABLE 1:** Summary of the number of viral evolutionary rate estimates used in this study

Viral group	Type of virus	No. of evolutionary rate estimates					
		Entire data set			Each data subset		
		Short-term rate	Long-term rate	Total	Short-term rate	Long-term rate	Total
Group I	dsDNA virus	9	12	21	3–8	1–5	7–10
Group II	ssDNA virus	45	2	47	9–12	1–2	11–13
Group IV	(+)ssRNA virus	122	1	123	45	1	46
Group V	(−)ssRNA virus	103	3	106	12–16	1	13–17
Group VI	RT-RNA virus	69	16	85	1–4	1–6	5–7
Group VII	RT-DNA virus	11	3	14	1	1	2
Total		359	37	396	71–85	6–15	84–95

These rate estimates are not phylogenetically independent, however, and this may bias our analyses if it is unaccounted for. To construct data subsets of phylogenetically independent rate estimates, we constrained the sampling criterion so that only one rate estimate was sampled per virus (defined by their common names [Table S1 in the supplemental material]), and all of its rates had the same probability of being sampled. We also noted that some of these rate estimates are still phylogenetically independent despite being for different viruses. For example, the short-term rates of herpes simplex virus 1 and varicella-zoster virus are phylogenetically independent, but both are phylogenetically nested within the long-term rates of alphaherpesviruses, which are, in turn, nested within those of alpha- and betaherpesviruses. In such cases, we divided the rates into phylogenetically independent groups while maximizing the number of rate estimates within each group. In this case, for instance, the rates were divided into three groups: (i) those of herpes simplex virus 1 and varicella-zoster virus, (ii) those of alphaherpesviruses, and (iii) those of alpha- and betaherpesviruses. For a particular data subset, only one of these groups was sampled with the same probability. Note that the number of rates may differ among groups, and thus, the number of rate estimates may differ across data subsets. Furthermore, the sampling was also constrained such that in each data subset, there was at least one short-term and one long-term rate estimate for each viral group. This constraint was imposed so that the rate decay dynamics could be estimated reliably. In total, 1,000 pseudoreplicated data subsets of phylogenetically independent rate estimates were constructed. See the sampling count in Table S1 in the supplemental material and the summary of the number of rate estimates for each data subset in Table 1.

### TDRP analyses at the level of Baltimore classification viral groups.

For each of the 1,000 pseudoreplicated data subsets, the rate estimates and measurement timescales were log-transformed (base 10) and were fitted to three linear models (equivalent to modeling a power law relationship between the rate estimates and measurement timescales): (i) rate estimate as a function of the timescale of rate measurement, (ii) rate estimate as a function of the timescale of rate measurement and viral group, and (iii) rate estimate as a function of the timescale of rate measurement and viral group, with separate rate decay slopes for each group. Model parameters were estimated by using the linear model (*lm*) function, implemented in R, version 3.1.2 (34). The significance levels of the estimated parameters were evaluated by *t* tests. Analyses of variance (ANOVA) and F tests were used to compare the models. Complete pairwise comparisons of the intercepts were performed by using the *glht* function, implemented in R, version 3.1.2 (34), to determine which viruses evolve faster or slower than others. In order to capture all of the parameter estimation uncertainties across 1,000 pseudoreplicated subanalyses, we constructed the distributions of parameter values by sampling 100 sets of parameter values from each of the 1,000 pseudoreplicated analyses and combined them as one to form an overall distribution (containing 100,000 sets of parameter estimates in total). Fisher's method was used to combine the *P* values from each subanalysis into a single overall *P* value. The results were evaluated at a significance level of 0.05.

### TDRP analyses at the level of viral genera.

A data set comprising rate estimates obtained from seven viral genera was assembled, including 4 rate estimates for the group I dsDNA virus genera Simplexvirus and Varicellovirus, 6 for the group II ssDNA virus genus Mastrevirus, 18 for the group IV (+)ssRNA virus genus Tobamovirus, 12 for the group V (−)ssRNA virus genus Hantavirus, 8 for the group VI RT-RNA virus genus Deltaretrovirus, and 3 for the group VII RT-DNA virus genus Avihepadnavirus. The Simplexvirus and Varicellovirus genera were combined as one data set, because there were not enough data points for separate analyses. The rate estimates and measurement timescales were log-transformed (base 10) and were fitted to three different linear models as described above. Parameter estimation, model comparison, and the construction of the distributions of estimated parameter values were also performed as described above.

### Sensitivity analyses.

Although we controlled for the rate measurement methods, some of the rate estimates, especially the short-term ones, might still be erroneous, since they are estimated from molecular data sets that do not contain sufficient temporal structure. We conducted simulations to evaluate how sensitive our analyses are to the presence of this specific type of erroneous rate estimate. We simulated 55 molecular sequences, each of which is 1,000 bp long. The evolutionary process was assumed to be a nonhomogeneous Poisson process, with the instantaneous rate of substitution (*r_t_*) being constant across sites but varying through time, governed by a power law decay function (*r_t_* = *r*_0_*t*^−β^). Guided by the results obtained from the TDRP analyses, the distribution of β (the rate decay slope) was assumed to be normal, with a mean of 0.65 and a standard deviation of 0.041. *r*_0_ (an apparent instantaneous evolutionary rate at one year before the present) was randomly sampled from a log-uniform distribution, ranging from 10^−4^ to 10^−2^ s/n/y. Fifty sequences were simulated to evolve over short timescales, spanning 10 to 1,000 years, and five sequences evolve over long timescales, ranging from 1,000 to 100 million years. The timescales were sampled from log-uniform distributions. The total numbers of substitutions were counted and were divided by their corresponding timescales to derive average substitution rate estimates. If the simulation resulted in at least one zero rate estimate, the simulation was rerun with a new sampled set of parameter values. To model erroneous rate estimates, a portion of short-term substitution numbers (20%, 40%, 60%, 80%, 100%) was shuffled before dividing by time, breaking the correlation between the two. The rate estimates and measurement timescales were log-transformed (base 10) and were fitted to a linear function using the *lm* function, implemented in R, version 3.1.2 (34). One hundred simulations were performed for each setting (0%, 20%, 40%, 60%, 80%, 100% erroneous short-term rate estimates). We then compared the distributions of the intercepts and slopes of the rate decay curves obtained from simulations containing erroneous rates to those obtained from the control simulation (0% erroneous rate estimates) using Wilcoxon signed-rank tests, implemented in R, version 3.1.2 (34). The *P* values were corrected by using the Bonferroni correction for multiple testing. In addition, we also performed sensitivity analyses exclusively on short-term rate estimates to investigate the effects of erroneous rates on the short-term TDRP analyses (<1,000 years).

### Short-term TDRP analyses.

Only the short-term rates of groups II, IV, and V were used, since there were <10 phylogenetically independent short-term rate estimates for groups I, VI, and VII in each data subset (Table 1). The rate estimates and measurement timescales were log-transformed (base 10) and were fitted to three different linear models as described for the overall TDRP analyses. Parameter estimation, model comparison, and the construction of the distributions of estimated parameter values were also conducted as described above.

### Estimating lentiviral evolutionary timescales by using our TDRP model.

A phylogeny of lentiviruses was estimated from a manually curated integrase nucleotide alignment (420 nucleotides [nt], 34 sequences) under the Bayesian phylogenetic framework by using BEAST, version 1.8.1 (35). We decided to use only the integrase region, because it is the only region currently available for Bioko drill SIV (SIVdrl-Bioko)—the sole lentivirus that provides the timescale information we used to calibrate our TDRP model (GenBank accession no. HM363427; see below). The alignment is available from the authors upon request. The Yule speciation process was applied, and the strict clock assumption with a fixed rate of 1 was used to estimate the total per lineage substitutions (*s*). Since we were interested only in the number of substitutions, and the phylogeny is not directly time calibrated, a strict molecular clock is sufficient. The best-fit nucleotide substitution models for each codon position, determined by jModelTest, version 2.1 (36), under the Akaike information criterion with a correction for finite sample sizes (AICc), were used [1st, GTR+I+Γ(4); 2nd, GTR+I+Γ(4); 3rd, TrN+Γ(4)]. African mainland drill SIV (SIVdrl) and SIVdrl-Bioko were constrained to be monophyletic. The Markov chain Monte Carlo (MCMC) was run for 50,000,000 steps. Parameters were logged every 5,000 steps, and the first 10% were discarded as burn-in. In total, 9,000 sets of posterior parameter estimates were obtained. Effective sample sizes of all parameters are >1,000, indicating that all parameters were well sampled and had converged.

We then simply converted the units of the branch lengths of each Bayesian posterior phylogeny from *s* to time (*t*) by using our TDRP model. The TDRP model states that log(*r̄*) **=** log(*s*/*t*) = α + β log(*t*), where “*r̄*” is the average evolutionary rate estimate (27); therefore, we can convert *s* to *t* simply by using the equation *t* = (*s*/10^α^)^1/(β + 1)^. Nonetheless, our results show that although the rate decay slopes (β) do not differ significantly among viruses, the intercepts (α) do. Thus, an α that is specific to lentiviruses is required in order to conduct these analyses.

To calculate α, which is equal to log(*s*) − (β + 1) log(*t*), we need β and at least a pair of corresponding *s* and *t* estimates. Based on the separation date of the African mainland and Bioko island, it has been proposed that SIVdrl and SIVdrl-Bioko separated >10,000 to 11,000 years ago (32), providing us with a timescale of the genetic divergence between SIVdrl and SIVdrl-Bioko. In this study, we assumed that the SIVdrl/SIVdrl-Bioko separation date is normally distributed, with a mean of 10,500 years and a standard error of (10,500 − 10,000) years/1.96, or (11,000 − 10,500) years/1.96, which equals 255.10 years. For each of the estimated Bayesian posterior trees, we first extracted the *s* estimate since the MRCA of SIVdrl and SIVdrl-Bioko to the present (*s*_cal_) and sampled one SIVdrl/SIVdrl-Bioko split date (*t*_cal_) from the assumed normal distribution. We then sampled one rate decay slope estimate (β) from the slope distribution, obtained from the overall TDRP analyses, and, in turn, computed the α of the lentivirus-specific TDRP model using the relationship α = log(*s*_cal_) − (β + 1) log(*t*_cal_). The calibrated model, *t* = (*s*/10^α^)^1/(β + 1)^, was then used to compute the *t* estimates of other nodes given their *s* estimates. This process was applied to all of the 9,000 posterior estimated trees in order to obtain the full posterior distributions of *t* estimates.

## RESULTS

### TDRP among Baltimore classification viral groups.

A total of 396 viral nucleotide substitution rate estimates were collected from 133 studies for TDRP analyses (Table 1; Fig. 1A; Table S1 in the supplemental material). The rates were sampled from all Baltimore classification viral groups except group III, since to our knowledge, long-term rate estimates (computed over timescales of >1,000 years) are not currently available for this group. We demonstrated previously that a simple power law curve can empirically describe the TDRP well for foamy viruses (27). We therefore use this model as the basis for our investigation. To do so, we log-transformed (base 10) the rate estimates (*r̄*) and their associated measurement timescales (*t*), and, in turn, fitted a linear model to them for statistical analyses; i.e., log(*r̄*) = α + β log(*t*) (equivalent to the equation *r̄* = 10^α^*t*^β^), where α is an intercept and β is a slope.

As shown previously (26, 27), our analyses suggested that viral evolutionary rate estimates are negatively correlated with their measurement timescales (*P* value of <0.05 in 1,000/1,000 subanalyses; combined *P* value, <0.001). Surprisingly, the slopes of the rate decay (β estimates) do not differ significantly among viral groups (*P* value of <0.05 in 27/1,000 subanalyses; combined *P* value, 0.133) and are estimated to be ∼−0.65 (95% highest probability density [95% HPD] = −0.72, −0.57); i.e., for every 3-fold increase in the measurement timescale, the value of the rate estimate decreases by approximately half (∼2.03×; 95% HPD = 1.86×, 2.21×).

Our analyses showed that, overall, the power law rate decay model can describe the data well (median *R*-squared value [*R*^2^] = 0.89; 95% HPD = 0.85, 0.93). We found that the rate estimates differ significantly among viral groups even when one adjusts for the rate decay dynamics (*P* value, <0.05 in 1,000/1,000 subanalyses; combined *P* value, <0.001). To determine the rate differences among viruses, the intercepts of the models (α estimates), which represent rate estimates that are controlled for the timescale of measurement (i.e., 10^α^ is equivalent to a rate that is estimated over a period of 1 year), were compared. The results show the following ranking of rates of viruses (expressed in units of s/n/y), from lowest to highest: group I dsDNA viruses (4.36 × 10^−4^; 95% HPD = 1.24 × 10^−4^, 1.48 × 10^−3^), group VII RT-DNA viruses (8.12 × 10^−4^; 95% HPD = 7.30 × 10^−5^, 9.82 × 10^−3^) and group VI RT-RNA viruses (1.12 × 10^−3^; 95% HPD = 2.71 × 10^−4^, 4.50 × 10^−3^) (approximately equal), group II ssDNA viruses (1.75 × 10^−3^; 95% HPD = 7.37 × 10^−4^, 4.09 × 10^−3^), group V (−)ssRNA viruses (6.87 × 10^−3^; 95% HPD = 3.11 × 10^−3^, 1.46 × 10^−2^) and group IV (+)ssRNA viruses (8.63 × 10^−3^; 95% HPD = 4.97 × 10^−3^, 1.45 × 10^−2^) (approximately equal) (Fig. 1B and C).

### TDRP within viral genera.

We noted that although the TDRP exists at the level of the Baltimore classification viral groups, it is possible that this might be merely an artifact, resulting from our tendency to use fast-evolving and slow-evolving viruses for short-term and long-term viral evolutionary studies, respectively. That is, it is possible that viral evolutionary rate estimates may in fact be independent of the rate measurement timescale at lower taxonomic levels, with some lineages evolving more slowly or more rapidly than others. However, due to our tendency to use fast- and slow-evolving viruses for short-term and long-term viral evolutionary studies, respectively, the TDRP may emerge as an artifact at the level of the Baltimore classification viral groups, in which all of these different rapidly and slowly evolving viruses were analyzed together.

To examine this possibility, we investigated the TDRP at the level of viral genera to see whether the rate decay pattern still holds or not. We performed the same TDRP analyses on seven viral genera sampled across six viral groups, including the group I dsDNA virus genera Simplexvirus and Varicellovirus, the group II ssDNA virus genus Mastrevirus, the group IV (+)ssRNA virus genus Tobamovirus, the group V (−)ssRNA virus genus Hantavirus, the group VI RT-RNA virus Deltaretrovirus, and the group VII RT-DNA virus genus Avihepadnavirus. These genera were chosen because the timescales of their rate measurement cover the longest time span (in term of orders of magnitude). The rates of simplexviruses and varicelloviruses were combined for analysis because there were not enough data points to be analyzed individually. Note that these rate estimates are not phylogenetically independent, and thus, the results should be interpreted with caution.

**FIG 2:**
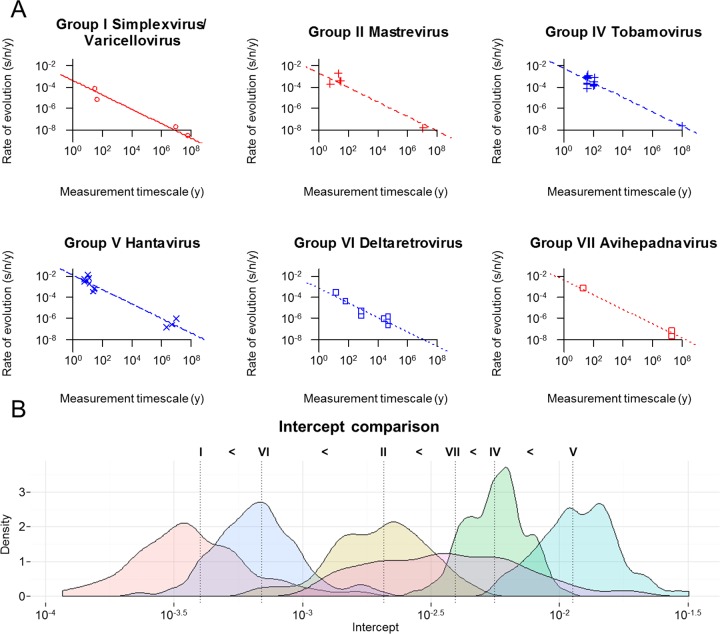
Time-dependent rate phenomenon among viral genera. (A) The lines represent the best fit models; open red circles and solid line, group I genera Simplexvirus and Varicellovirus; red plus signs and dashed line, group II genus Mastrevirus; blue plus signs and dashed line, group IV genus Tobamovirus; blue crosses and long-dashed line, group V genus Hantavirus; open blue squares and dotted line, group VI genus Deltaretrovirus; open red squares and dotted line, group VII genus Avihepadnavirus. (B) Complete pairwise comparisons of the intercepts of the rate decay curves (representing rate estimates controlled for a 1-year timescale of rate measurement). Vertical dotted lines indicate median estimates. The differences among intercepts were evaluated at a significance level of 0.05.

Our analyses show that the model can describe the data well (*R*^2^ = 0.95), and that the TDRP still holds even at the level of viral genera (*P* value, <0.001). The slopes of the rate decay curves do not differ significantly among the genera (*P* value, 0.89) and are estimated to be ∼−0.68 (95% HPD = −0.74, −0.62). Remarkably, this slope estimate is strikingly similar to the slope estimate we obtained from the TDRP analyses at the level of viral groups (∼−0.65; 95% HPD = −0.72, −0.57). These findings further support our observation that the rate decay slopes do not differ significantly among viruses and suggest, simultaneously, that the TDRP at the level of viral groups is not an artifact emerging from systematic biases in our tendency to use fast- and slow-evolving viruses for short-term and long-term evolutionary studies, respectively. Moreover, this finding also suggests that the problem of phylogenetic nonindependency, although present in our data set, does not greatly affect the analyses. By comparing the intercepts of the models, we found that different viral genera evolve at different rates (*P* value, <0.001), which may be ranked as follows (expressed in units of s/n/y), from lowest to highest: the group I genus Simplexvirus/Varicellovirus (3.99 × 10^−4^; 95% HPD = 1.28 × 10^−4^, 1.24 × 10^−3^), the group VI genus Deltaretrovirus (6.94 × 10^−4^; 95% HPD = 3.05 × 10^−4^, 1.58 × 10^−3^), the group II genus Mastrevirus (2.07 × 10^−3^; 95% HPD = 8.93 × 10^−4^, 4.82 × 10^−3^), the group VII genus Avihepadnavirus (3.92 × 10^−3^; 95% HPD = 1.04 × 10^−3^, 1.47 × 10^−2^), the group IV genus Tobamovirus (5.57 × 10^−3^; 95% HPD = 3.26 × 10^−3^, 9.52 × 10^−3^), and the group V genus Hantavirus (1.13 × 10^−2^; 95% HPD = 5.88 × 10^−3^, 2.16 × 10^−2^) (Fig. 2). These results are largely consistent with the results obtained from the TDRP analyses at the level of viral groups.

### Sensitivity analyses.

Despite the control of the rate measurement methods, it is likely that some of the rate estimates may still be erroneous, especially the short-term rate estimates, computed from data sets that do not contain sufficient temporal structure. In such a scenario, it is expected that the number of substitutions (*s*) and *t* are independent of one another [i.e., *s*(*t*) = *c*, where *c* is a constant], and thus, the relationship between *r̄* and *t* is expected to be as follows: *r̄* = *s*/*t* = *c*/*t*; i.e., log(*r̄*) = log(*c*) − log (*t*). Therefore, the presence of erroneous short-term rate estimates can systematically bias the rate decay slope toward −1.

To evaluate the effects of these erroneous rate estimates on our analyses, we simulated 50 short-term and 5 long-term substitution rate estimates under the TDRP model and replaced 20%, 40%, 60%, 80%, and 100% of the short-term rates with erroneous rates, calculated from noncorrelated substitution numbers and timescales (see Materials and Methods). The intercepts and slopes of the rate decay curves were compared to those obtained from the control simulation, in which all of the rates were simulated under the TDRP model.

**FIG 3:**
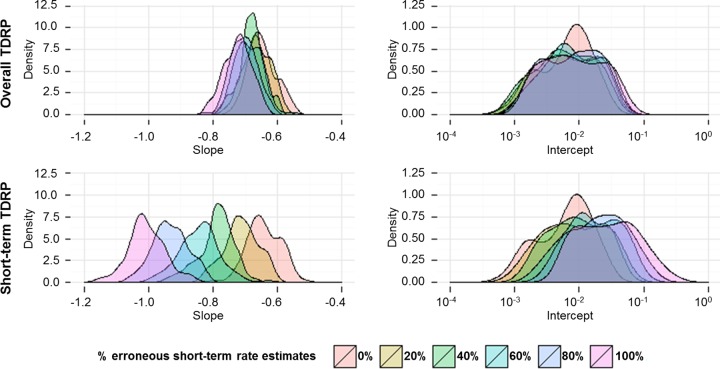
Effects of erroneous short-term rate estimates derived from noncorrelated substitution numbers and timescales of rate measurement on the rate decay curve inference. Simulations were used to examine how the presence of erroneous short-term rate estimates may bias the time-dependent rate phenomenon (TDRP) analyses, assuming a nonhomogeneous Poisson evolutionary process and a power law rate decay curve. Left and right graphs show slope and intercept estimates, respectively, of the rate decay curve, computed in the presence of 20%, 40%, 60%, 80%, and 100% erroneous short-term rate estimates. Top and bottom graphs show how the erroneous short-term rates affect overall and short-term TDRP curves, respectively. The intercept and slope of the rate decay curve obtained from control simulations (0% erroneous rate estimates) were used as controls.

Our analyses reveal that the intercepts obtained from all simulations largely overlap (Fig. 3, top right). The intercept estimate (expressed in units of s/n/y) was 7.02 × 10^−3^ (95% HPD = 1.08 × 10^−3^, 2.41 × 10^−2^) with 0% erroneous short-term rate estimates, 6.09 × 10^−3^ (95% HPD = 1.12 × 10^−3^, 3.25 × 10^−2^) with 20%, 6.05 × 10^−3^ (95% HPD = 8.96 × 10^−4^, 2.89 × 10^−2^) with 40%, 7.03 × 10^−3^ (95% HPD = 1.43 × 10^−3^, 4.01 × 10^−2^) with 60%, 8.43 × 10^−3^ (95% HPD = 1.68 × 10^−3^, 4.19 × 10^−2^) with 80%, and 7.65 × 10^−3^ (95% HPD = 1.61 × 10^−3^, 4.46 × 10^−2^) with 100%. In fact, we could not detect biases in the intercept estimates even when all of the short-term rates were erroneous (when intercept estimates were compared against the controlled intercept by the Wilcoxon test, *P* values were >0.999 with 20% erroneous short-term rate estimates, >0.999 with 40%, 0.632 with 60%, 0.705 with 80%, and 0.428 with 100%). We estimated the slopes to be −0.65 (95% HPD = −0.71, −0.55) with 0% erroneous short-term rate estimates, −0.66 (95% HPD = −0.74, −0.59) with 20%, −0.68 (95% HPD = −0.75, −0.60) with 40%, −0.69 (95% HPD = −0.76, −0.62) with 60%, −0.71 (95% HPD = −0.78, −0.63) with 80%, and −0.72 (95% HPD = −0.81, −0.64) with 100% (Fig. 3, top left). The bias showed only when there were >20% erroneous rate estimates (*P* values were <0.001 with 40% to 100% erroneous rate estimates); otherwise, it could not be detected (*P* value was 0.142 with 20% erroneous rate estimates). Furthermore, even when the bias could be detected, the effect was very small. For example, with 60% erroneous rate estimates, the slope only changes by ∼0.04 with respect to the slope obtained from the control simulation, changing from ∼−0.65 (95% HPD = −0.71, −0.55) to ∼−0.69 (95% HPD = −0.76, −0.62). Even in the case of 100% erroneous short-term rates, the biased slope distribution still largely overlaps with that obtained from the control simulation. Combined, these results suggest that our TDRP analyses are extremely robust against erroneous rates derived from data sets containing insufficient temporal structure.

We also performed sensitivity analyses exclusively on short-term rate estimates. We found that in this case, erroneous short-term rate estimates can severely bias the TDRP curve. As expected, as the number of erroneous rate estimates increased, the slope was increasingly biased toward −1 (Fig. 3, bottom left). Slope estimates were −0.71 (95% HPD = −0.82, −0.62) with 20% erroneous short-term rate estimates, −0.78 (95% HPD = −0.92, −0.71) with 40%, −0.84 (95% HPD = −0.97, −0.73) with 60%, −0.94 (95% HPD = −1.02, −0.83) with 80%, and −1.01 (95% HPD = −1.10, −0.87) with 100% , all of which are significantly different from the controlled slope (−0.65; 95% HPD = −0.73, −0.55; *P* values were <0.001 for all comparisons). The intercept also became more and more overestimated (Fig. 3, bottom right). The intercept estimate (expressed in units of s/n/y) was 6.88 × 10^−3^ (95% HPD = 9.56 × 10^−4^, 2.48 × 10^−2^) with 0% erroneous short-term rates, 7.34 × 10^−3^ (95% HPD = 1.37 × 10^−3^, 4.10 × 10^−2^) with 20%, 9.24 × 10^−3^ (95% HPD = 2.14 × 10^−3^, 6.23 × 10^−2^) with 40%, 1.36 × 10^−2^ (95% HPD = 2.39 × 10^−3^, 6.98 × 10^−2^) with 60%, 2.12 × 10^−2^ (95% HPD = 3.91 × 10^−3^, 1.14 × 10^−1^) with 80%, and 2.86 × 10^−2^ (95% HPD = 5.37 × 10^−3^, 1.63 × 10^−1^) with 100%. When the intercept estimates were compared with the controlled intercept, the *P* value was 0.350 with 20% erroneous rate estimates, 0.015 with 40%, and <0.001 with 60% to 100%.

### Short-term TDRP.

**FIG 4:**
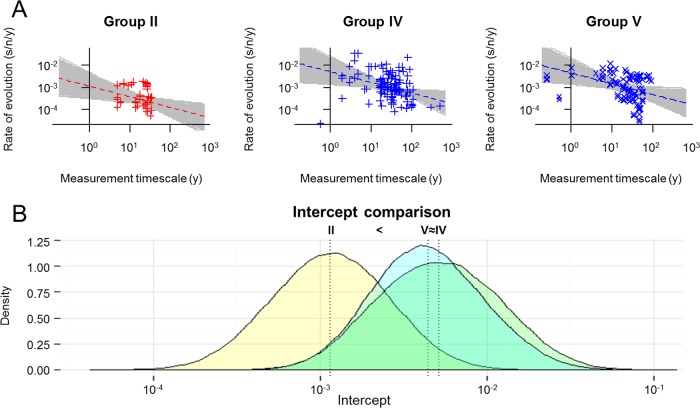
Short-term time-dependent rate phenomenon among viral groups. (A) Gray lines represent 1,000 individual best-fit models, where the slopes are the same across all viral groups. Red or blue lines represent models that are parameterized by median parameter estimates: red plus signs and dashed line, group II ssDNA viruses; blue plus signs and dashed line, group IV (+)ssRNA viruses; blue crosses and long-dashed line, group V (−)ssRNA viruses. (B) Complete pairwise comparisons of the intercepts of the rate decay curves (representing rate estimates controlled for a 1-year timescale of rate measurement). Vertical dotted lines indicate median estimates. The differences among intercepts were evaluated at a significance level of 0.05.

We also performed TDRP analyses exclusively on the short-term rate estimates in order to examine whether or not the pattern we observed in the overall TDRP analyses still holds. Groups I, VI, and VII were not included, however, because there were only strictly less than 10 phylogenetically independent short-term rates for these groups in each data subset (Table 1). Again, our analyses showed that the short-term rates are negatively correlated with their measurement timescales (*P* value, <0.05 in 840/1,000 subanalyses; combined *P* value, <0.001). The slope was estimated to be −0.49, slightly greater than the slope obtained from the overall TDRP analyses (−0.65; 95% HPD = −0.72, −0.57), with a much greater uncertainty (95% HPD = −0.93, −0.06). Consistent with this is a low *R*^2^ score of 0.26 (95% HPD = 0.10, 0.41). This is expected, however, since the variance of the rate estimates is extremely high with respect to the narrow range of the rate measurement timescales (Fig. 4). In fact, it is very striking that the rate decay pattern could still be observed even over a timescale of <1,000 years. Our analyses show that the short-term rate decay slopes do not differ significantly among viral groups (*P* value of <0.05 in 24/1,000 subanalyses; combined *P* value, 0.656). The temporally adjusted rate estimates (expressed in units of s/n/y) are also clearly different among viruses (*P* value of <0.05 in 963/1,000 subanalyses; combined *P* value, <0.001); those of group II ssDNA viruses (1.16 × 10^−3^; 95% HPD = 2.35 × 10^−4^, 5.36 × 10^−3^) are less than those of group V (−)ssRNA viruses (4.34 × 10^−3^; 95% HPD = 1.02 × 10^−3^, 2.02 × 10^−2^), which are approximately equal to those of group IV (+)ssRNA viruses (5.14 × 10^−3^; 95% HPD = 1.03 × 10^−3^, 2.54 × 10^−2^) (Fig. 4). These findings are consistent with the results obtained from the overall TDRP analyses.

### Case study: reestimating the time scale of evolution of lentiviruses.

Lentiviruses are a group of medically important viruses whose evolutionary history has been studied extensively. Nevertheless, there is still little consensus regarding when lentiviruses originated. In fact, estimating the origin of lentiviruses from extant viral molecular sequences has long been one of the most difficult challenges in lentiviral evolutionary biology. For example, while paleovirological analyses strongly support an age of millions of years for SIVs (28, 29), all previous standard molecular analyses of modern SIVs consistently estimated their age to be at most tens of thousands of years old (8, 30–32). This is likely due to the fact that the TDRP was not accounted for in previous molecular analyses. Indeed, the TDRP has been noticed in lentiviruses (26), but their TDRP-corrected age estimate has not been inferred. Here we illustrate the use of our TDRP model by reestimating the time scale of lentiviruses.

**FIG 5:**
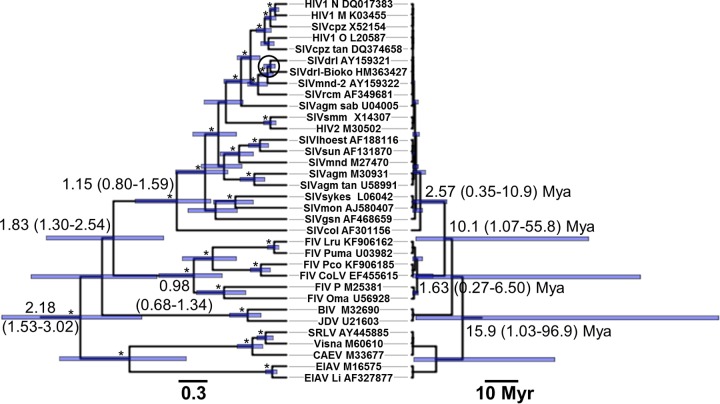
Lentivirus phylogeny and evolutionary timescale. (Left) Maximum clade credibility phylogeny of lentiviruses. The tree was estimated in the Bayesian phylogenetic framework under a strict clock assumption with a fixed rate of 1. The branch lengths and scale bar are in units of substitutions per site. The numbers on nodes are node heights in units of substitutions per site. The corresponding 95% highest posterior density intervals (HPDs) are given in parentheses. Asterisks indicate nodes with posterior support of >0.85. The split between SIVdrls and SIVdrl-Bioko (>10,000 to 11,000 years ago), which was used to calibrate the lentivirus-specific TDRP model, is circled. (Right) Time-calibrated lentivirus tree. The numbers on nodes are node heights in units of years before the present, inferred by using our TDRP model. Corresponding 95% HPDs are given in parentheses. The branch lengths and scale bar are also in units of millions of years (Myr).

We first estimated a phylogeny of extant lentiviruses from their integrase coding regions under the Bayesian phylogenetic framework and a strict molecular clock assumption (Fig. 5). The rate was fixed to 1, and thus, the node heights were expressed in units of substitutions per site. We then simply converted the units of branch lengths from substitutions per site to times by using our TDRP model. The rate decay slope was sampled from the slope we obtained from the overall TDRP analyses. The lentivirus-specific intercept was calibrated by using the separation date of African mainland drill SIVs (SIVdrl) and Bioko drill SIVs (SIVdrl-Bioko), which was >10,000 to 11,000 years ago (32) (see Materials and Methods).

The intercept of the lentivirus-specific TDRP curve was calculated to be ∼−2.20 (95% HPD = −2.54, −1.83). This result implies that the short-term rate of lentiviruses (calculated over a 1-year period) is ∼6.34 × 10^−3^ (95% HPD = 2.86 × 10^−3^, 1.48 × 10^−2^) s/n/y, comparable to the established short-term rate of lentiviral evolution (10, 37–40). We then used this model to compute the timescales of other nodes (Fig. 5). Note that since the curve is calibrated by a geographical separation date, which likely postdates the actual SIVdrl/SIVdrl-Bioko speciation date (32), our lentiviral evolutionary time scale estimates should be interpreted as lower-bound estimates.

Our analyses suggest that lentiviruses as a whole have an ancient origin and are >15.9 (95% HPD = 1.03, 96.9) million years (Myr) old. We also estimated SIVs to be >2.57 (95% HPD = 0.35, 10.9) Myr old, feline immunodeficiency viruses (FIVs) to be >1.63 (95% HPD = 0.27, 6.50) Myr old, and the MRCA of SIVs and FIVs to be >10.1 (95% HPD = 1.07, 55.8) Myr old. The uncertainties of the age estimates are large, however, spanning ∼2 orders of magnitude. We believe that this is caused partly by the fact that we used a short nucleotide alignment in our analyses (integrase [420 nt]). Our alignment was limited to the integrase region, since this is the only region currently available for SIVdrl-Bioko—the only lentivirus that provides the timescale calibration information in our analyses. Note that, in our case, expanding the alignment would not alleviate the problem. This is because the SIVdrl-Bioko sequence would still be mostly blank, and thus, the uncertainty of its substitution estimate, with which the TDRP model was calibrated, would still be high. Furthermore, expanding the alignment would likely bias the mean estimates of the timescales as well. A longer sequence of SIVdrl-Bioko is required to overcome this particular problem and, in turn, to increase the precision of the date estimates.

## DISCUSSION

The last decade or so has seen several studies and reviews reporting and discussing the discrepancies among viral rate estimates that are calculated over different time frames (24, 26, 27, 32, 41, 42). The recurring theme is that, as can be seen in Fig. 1A, viral short-term rate estimates (typically calculated from heterochronous molecular data sets) tend to be much greater than long-term rate estimates (usually inferred based on the virus-host cospeciation assumption or on geographical separation dates). Indeed, it has been observed that viral evolutionary rate estimates are systematically negatively correlated with the time scale of rate estimation, continuously decreasing as the measurement time scale increases (25–27). Duchêne et al. (26) demonstrated this phenomenon in both RNA and DNA viruses by fitting separate regressions onto them *a priori*, and analyses of foamy viruses indicate that this phenomenon can be empirically described very well by a power law curve (27). In this study, we used the power law model as our basis for further investigation of this time-dependent rate phenomenon (TDRP) at a finer taxonomic scale, across different Baltimore classification viral groups and genera. We also statically compared how the TDRP dynamics of different viruses differ from one another.

We found that the TDRP holds both at the level of viral groups (Fig. 1) and at the level of genera (Fig. 2) and that the power law models can describe the phenomenon well, as indicated by high *R*^2^ scores. Our sensitivity analyses show that overall, the inference of the rate decay curve is extremely robust against erroneous short-term rate estimates, derived from data sets with weak temporal signals (Fig. 3, top). This robustness likely comes from the fact that the sampling times of the short-term rates are relatively short (<1,000 years) compared to the timescales of the overall TDRP examination (100 Myr). This result also suggests that the difference between short-term and long-term rate estimates is likely the main factor that drives the inference of the overall TDRP slope. This robustness, however, raises a particular concern, which is that the TDRP we observed here could be driven by only a few long-term rate estimates and, in the worst-case scenario, that the majority of the short-term rates we used in our analyses are erroneous, calculated from data sets with no temporal signal. Nevertheless, our sensitivity analyses show that erroneous short-term rates can severely bias the short-term TDRP curve estimation (Fig. 3, bottom). However, we found that the TDRP could still be detected even among short-term rate estimates and that the results from the short-term TDRP analyses are all consistent with those obtained from the overall TDRP analyses (Fig. 4). This indicates a temporal structure for the short-term rate estimates and suggests that our analyses likely contain only a few erroneous short-term rate estimates, if any. This finding also shows that our overall TDRP analyses were not driven solely by a few long-term rate estimates and that the rate decay slope is indeed stable across all timescales.

Many factors have been put forward as underlying causes of the TDRP (for a review, see reference 25). Nevertheless, the fact that the TDRP can be found across all viruses suggests that it is a result of natural phenomena that all viruses encounter. There are a wide range of evolutionary scenarios that all viruses are expected to encounter, which can lead to depressed long-term substitution rate estimates and inflated short-term rate estimates, and hence have the potential to explain the TDRP. For example, despite their large diversity in biological features, life histories, and replication strategies, all viruses experience repeated bottlenecking transmission events, which can lead to the accumulation of deleterious mutations within viral genomes (43–45). It is also clear that all viruses have highly subdivided population structures. This means that slightly defective viruses within one subpopulation (e.g., within a host individual or a group of individuals within a transmission network) might not be immediately outcompeted by their slightly fitter relatives in different subpopulations, exacerbating the accumulation of deleterious mutations (46, 47). It is therefore expected that deleterious mutations can persist within viruses (as a species) for significant amounts of time, causing rates estimated over short timescales (i.e., toward the present) to be systematically overestimated. In contrast, the long-term rate of viral evolution is a product of coevolution between viruses and their slowly evolving hosts, constrained by many molecular features needed for proper biological functions and host interaction, such as overlapping reading frames, secondary structure, and regions with multiple functions (48). These strong evolutionary constraints are expected to result in extensive site saturation and convergent evolution (48). This, in turn, can lead to systematically underestimated long-term rate estimates. Overall, given that the TDRP is a prevalent evolutionary feature of viruses, we hypothesize that the bottlenecking nature of viral transmission, highly subdivided population structure, and substitution saturation are the main drivers of the TDRP.

Surprisingly, all of our analyses reveal that the speeds at which the rates decay do not differ significantly among viruses despite their vast diversity in replication strategies, molecular features, and evolutionary histories. This uniformity of the rate decay slopes suggests that, irrespective of their biological and molecular features, all viruses may experience the same degrees of population subdivision and bottlenecking. Moreover, this finding may also suggest that the degree of substitution saturation might be the same across all viruses. The most parsimonious form of this hypothesis predicts that all viral genomes might have similar proportions of conserved, neutral, and adaptive sites. Indeed, it has been shown that poor modeling of rate heterogeneity among sites can cause rate estimates to appear to be time dependent (49). It is therefore possible that the TDRP might be caused partially by the proportions of conserved, neutral, and adaptive sites being poorly modeled by our current methods. Examination of these hypotheses will require experiments that elucidate how population subdivision, bottlenecking, and the proportions of conserved, neutral, and adaptive sites affect the measurement of evolutionary rates.

We want to emphasize that the TDRP is likely only an apparent phenomenon—that the actual rate of substitution fixation within viral species is time independent but appears to vary over time, likely due to the presence of transient deleterious mutations (which inflate short-term rate estimates) and substitution saturations (which deflates long-term rate estimates) (24, 25, 27). Lying between the two extreme ends of short-term/long-term rate estimates is an effective (apparent) rate that matches the true time-independent rate of viral evolution, resulting from a balance between the presence of site saturations and transient deleterious mutations within viral genomes. It is likely that these true rates of viral evolution differ between viral lineages; after all, the replication strategies, molecular characteristics, and evolutionary histories of these highly distinct viruses are drastically different, and these should in some ways influence their (actual) substitution rates. On the other hand, it is expected that viruses with similar biology and molecular features should have similar evolutionary rates. It is therefore surprising to see emerging evidence showing that the difference in viral evolutionary rates is extremely large even among viruses of the same type (11, 12) and that the boundary between the rates of DNA and RNA viruses is very blurry to the point that there may be no strict division between the two (13, 14). Our analyses show, for the first time, that the TDRP can explain a large portion of this variance, and the boundary between the rates of different viruses becomes more apparent once the TDRP is accounted for.

Our analyses show that our TDRP model can account for ∼90% of the variance within the rate estimates (*R*^2^, 0.89 for TDRP among viral groups and 0.95 for TDRP among viral genera), and after adjusting for the TDRP, we found that dsDNA viruses evolve more slowly than ssDNA viruses and reverse transcribing (RT) viruses, which, in turn, evolve more slowly than ssRNA viruses consistently across all analyses (Fig. 1C and 2B). These findings are consistent with the experimental observations that (i) DNA is more chemically stable than RNA (50), (ii) single-stranded nucleic acids have higher instability than double-stranded nucleic acids (51), and (iii) the replication fidelity of DNA polymerase is higher than that of reverse transcriptase, which, in turn, is higher than that of RNA polymerase (13, 52). Our results also greatly resemble the observations that the mutation rates of dsDNA viruses are lower than those of ssDNA viruses and RT viruses, which, in turn, are lower than those of RNA viruses (11, 53). Together, our findings support the notion that there indeed exists a clear basic division between the rates of evolution of dsDNA viruses, ssDNA/RT viruses, and ssRNA viruses and that this can be explained very well by differences in molecular biology among viruses.

One of the most important implications of the TDRP is that naïvely extrapolating evolutionary rate estimates across different timescales for evolutionary inferences can severely bias the analyses (26, 27). One of the best-known cases of this is perhaps the severe underestimation of lentiviral evolutionary timescales by standard phylogenetic analyses. Our TDRP model may offer a partial solution to this problem, allowing us to reconcile the long-term and short-term viral evolutionary studies. To illustrate this, we used our model to reestimate the ages of various lentiviral groups (Fig. 5).

Our analyses estimate that lentiviruses are millions of years old, and the date estimates are highly consistent with paleovirological evidence and the knowledge of lentiviral host migration. SIVs were previously estimated to be at most tens of thousands of years old by phylogenetic analyses that did not account for the TDRP (32). Our TDRP model, on the other hand, estimated SIVs to be >2.57 (95% HPD = 0.35, 10.9) Myr old. Compared to previous age estimates, our estimate fits much better with paleovirological evidence that suggests that SIVs are many millions of year old (28). We also estimated FIVs to be >1.63 (95% HPD = 0.27, 6.50) Myr old. Based on the geographical distribution of FIVs, Troyer et al. (54) proposed that modern FIVs likely originated before the last time felids crossed the Bering Strait, in the late Pliocene (>2.58 Myr ago) to early Pleistocene (>1.80 Myr ago) epoch (55). Our estimate fits very well with this knowledge of felid migration. Furthermore, previous phylogenetic analyses revealed that a gray mouse lemur endogenous lentivirus (pSIVgml) is embedded within the clade of SIVs and FIVs (29), and it is >4 Myr old (56). This places a lower-bound estimate of the time to the MRCA (tMRCA) of SIVs and FIVs at 4 Myr. Again, our result is consistent with this finding, suggesting that the tMRCA of SIVs and FIVs is >10.1 (95% HPD = 1.07, 55.8) Myr. Last, we estimated lentiviruses as a whole to be >15.9 (95% HPD = 1.03, 96.9) Myr old. This is congruent with the results from analyses of orthologous rabbit-hare endogenous lentiviruses, which suggested that lentiviruses are >12 Myr old overall (57).

It is also noteworthy that, based on the observed basal phylogenetic placement of pSIVgml within SIVs, an ancient cospeciation between primates and lentiviruses has also been proposed, implying that primate lentiviruses are 85 Myr old (29). Our estimated tMRCA for SIVs and FIVs, at 10.1 Myr, is much more recent than that date. However, it should be noted that this date estimate is a lower-bound estimate, since our model was calibrated with a lower-bound viral speciation date. Furthermore, given the limited number of basal viral lineages in our analyses, it is possible that the number of substitutions of basal branches, and therefore deep evolutionary timescales, might still be significantly underestimated due to site saturation. We therefore consider that it is too early to reject this hypothesis. Further investigation with more basal lentiviral sequences may resolve this conundrum.

Overall, our results support the predictive value of our TDRP model and suggest that our model is useful as a guideline for further improvement of current evolutionary analytical tools. To the best of our knowledge, this is the first demonstration of an ancient origin of lentiviruses by phylogenetic analyses of extant viruses, closing the gap between ancient and recent viral evolution. Nonetheless, we note the large uncertainties of our lentiviral age estimates, spanning ∼2 orders of magnitude (Fig. 5). Rather than representing intrinsic problems of the model, we believe that, in addition to a short nucleotide alignment, this highlights the inherent difficulties in working with the small number of data points available for calibrating the model and extrapolating rate estimates across large timescales. This problem could be partially overcome by developing a TDRP model under a full Bayesian or maximum likelihood phylogenetic framework. Such a model would allow us to fully integrate and control the prior distributions of the rates, timescales, and substitutions, as well as to jointly estimate the parameters, thereby increasing precision.

## Supplementary Material

Supplemental material
