# Supplementary material for: Time-Dependent Rate Phenomenon in Viruses
Source: J Virol. 2016 Jul 27;90(16):7184–95. doi: 10.1128/JVI.00593-16 (PMC4984659; doi:10.1128/JVI.00593-16)
Supplement: Supplemental material [file supp_90_16_7184__index.html]

Time-Dependent Rate Phenomenon in Viruses — Supplemental material 

# Time-Dependent Rate Phenomenon in Viruses

## Supplemental material

- Supplemental file 1 -

  Table S1 (Viral nucleotide substitution rate estimates.)

  References

  PDF, 913K
