# Supplementary material for: Time-Dependent Rate Phenomenon in Viruses
Source: J Virol. 2016 Jul 27;90(16):7184–95. doi: 10.1128/JVI.00593-16 (PMC4984659; doi:10.1128/JVI.00593-16)
Supplement: Supplemental material [file JVI.00593-16_zjv999181836so1.pdf]

Table S1. Viral nucleotide substitution rate estimates

Viral group	Viral family	Viral genus	Common name	Rate estimate value (s/n/y)	Time-scale (year)	Alignment	Rate estimation method	Ref	Sampling count
**Short-term rate estimates**									
Group I/dsDNA virus	Adenoviridae	Mastadenovirus	Human adenovirus B	7.20E-5	24	Hexon coding region	Tip-date calibration under the Bayesian phylogenetic framework	(1)	1000
Group I/dsDNA virus	Adenoviridae	Mastadenovirus	Human adenovirus C	3.46E-5	21	Hexon coding region	Tip-date calibration under the Bayesian phylogenetic framework	(1)	1000
Group I/dsDNA virus	Herpesviridae	Simplexvirus	Human simplex virus 1 (nested in alpha herpesvirus, and alpha and beta herpesvirus)	8.21E-5	27	Thymidine kinase coding region	Tip-date calibration under the Bayesian phylogenetic framework	(1)	155
Group I/dsDNA virus	Herpesviridae	Varicellovirus	Varicella zoster virus (nested in alpha herpesvirus, and alpha and beta herpesvirus)	6.26E-6	37	Genome	Tip-date calibration under the Bayesian phylogenetic framework	(1)	335
Group I/dsDNA virus	Papillomaviridae	Alphapapillomavirus	Human papillomavirus 16 (nested in papillomavirus)	3.94E-3	3	L1 protein coding region	Tip-date calibration under the Bayesian phylogenetic framework	(1)	510
Group I/dsDNA virus	Polyomaviridae	Orthopolyomavirus	Human polyomavirus JC	1.70E-5	33	Genome	Tip-date calibration under the Bayesian phylogenetic framework	(2)	472
Group I/dsDNA virus	Polyomaviridae	Polyomavirus	BK virus	4.34E-5	29	Genome (coding region)	Tip-date calibration under the Bayesian phylogenetic framework	(1)	1000
Group I/dsDNA virus	Poxviridae	Orthopoxvirus	Variola virus	9.32E-6	36	Genome	Tip-date calibration under the Bayesian phylogenetic framework	(1)	330
Group I/dsDNA virus	Poxviridae	Orthopoxvirus	Variola virus	1.7E-6	400	102 conserved genes	Internal-node calibration under the maximum-likelihood phylogenetic framework	(3)	350
Group II/ssDNA virus	Circoviridae	Circovirus	Porcine circovirus 2	1.44E-3	27	Coat protein coding region	Tip-date calibration under the Bayesian phylogenetic framework	(4)	100
Group II/ssDNA virus	Circoviridae	Circovirus	Porcine circovirus 2	1.21E-3	27	Genome	Tip-date calibration under the Bayesian phylogenetic framework	(4)	97
Group II/ssDNA virus	Circoviridae	Circovirus	Porcine circovirus 2	1.32E-3	27	ORF3	Tip-date calibration under the Bayesian phylogenetic framework	(4)	104
Group II/ssDNA virus	Circoviridae	Circovirus	Porcine circovirus 2	1.03E-3	27	Replicase coding region	Tip-date calibration under the Bayesian phylogenetic framework	(4)	108
Group II/ssDNA virus	Circoviridae	Circovirus	Porcine circovirus 2	8.74E-4	27	Replicase coding region-ORF3	Tip-date calibration under the Bayesian phylogenetic framework	(4)	92
Group II/ssDNA virus	Circoviridae	Circovirus	Porcine circovirus 2a (nested in porcine circovirus 2)	1.64E-3	25	Genome	Tip-date calibration under the Bayesian phylogenetic framework	(4)	499
Group II/ssDNA virus	Circoviridae	Circovirus	Porcine circovirus 2b (nested in porcine circovirus 2)	1.15E-3	9	Genome	Tip-date calibration under the Bayesian phylogenetic framework	(4)	499
Group II/ssDNA virus	Geminiviridae	Begomovirus	African cassava mosaic disease-causing virus (nested in South American begomovirus)	3.45E-4	5	BC1 coding region	Tip-date calibration under the Bayesian phylogenetic framework	(5)	80

Group II/ssDNA virus	Geminiviridae	Begomovirus	African cassava mosaic disease-causing virus (nested in South American begomovirus)	1.37E-3	7	Coat protein coding region	Tip-date calibration under the Bayesian phylogenetic framework	(5)	56
Group II/ssDNA virus	Geminiviridae	Begomovirus	African cassava mosaic disease-causing virus (nested in South American begomovirus)	1.60E-3	7	DNA-A segment	Tip-date calibration under the Bayesian phylogenetic framework	(5)	67
Group II/ssDNA virus	Geminiviridae	Begomovirus	African cassava mosaic disease-causing virus (nested in South American begomovirus)	1.33E-4	5	DNA-B segment	Tip-date calibration under the Bayesian phylogenetic framework	(5)	64
Group II/ssDNA virus	Geminiviridae	Begomovirus	African cassava mosaic disease-causing virus (nested in South American begomovirus)	2.77E-4	5	Nuclear shuttle protein coding region	Tip-date calibration under the Bayesian phylogenetic framework	(5)	72
Group II/ssDNA virus	Geminiviridae	Begomovirus	African cassava mosaic disease-causing virus (nested in South American begomovirus)	1.24E-3	5	Replication-associated protein coding region	Tip-date calibration under the Bayesian phylogenetic framework	(5)	83
Group II/ssDNA virus	Geminiviridae	Begomovirus	African cassava mosaic disease-causing virus (nested in South American begomovirus)	1.56E-3	7	Ori-C1-C4	Tip-date calibration under the Bayesian phylogenetic framework	(6)	82
Group II/ssDNA virus	Geminiviridae	Begomovirus	Tomato yellow leaf curl virus (nested in South American begomovirus)	4.04E-4	18	Ori-C1-C4	Tip-date calibration under the Bayesian phylogenetic framework	(6)	89
Group II/ssDNA virus	Geminiviridae	Begomovirus	Tomato yellow leaf curl virus (nested in South American begomovirus)	4.63E-4	18	Coat protein coding region	Tip-date calibration under the Bayesian phylogenetic framework	(7)	113
Group II/ssDNA virus	Geminiviridae	Begomovirus	Tomato yellow leaf curl virus (nested in South American begomovirus)	2.88E-4	18	Genome	Tip-date calibration under the Bayesian phylogenetic framework	(7)	90
Group II/ssDNA virus	Geminiviridae	Begomovirus	Tomato yellow leaf curl virus (nested in South American begomovirus)	1.37E-3	11	Intergenic region	Tip-date calibration under the Bayesian phylogenetic framework	(7)	105
Group II/ssDNA virus	Geminiviridae	Begomovirus	Tomato yellow leaf curl virus (nested in South American begomovirus)	1.75E-3	12	Intergenic region	Tip-date calibration under the Bayesian phylogenetic framework	(7)	107
Group II/ssDNA virus	Geminiviridae	Mastrevirus	Maize streak virus	3.87E-4	29	Ori-C1-C4	Tip-date calibration under the Bayesian phylogenetic framework	(6)	320
Group II/ssDNA virus	Geminiviridae	Mastrevirus	Maize streak virus	2.0E-4	6	Genome	Tip-date calibration under the Bayesian phylogenetic framework	(8)	381
Group II/ssDNA virus	Geminiviridae	Mastrevirus	Maize streak virus	2.1E-4	6	Genome	Tip-date calibration under the Bayesian phylogenetic framework	(8)	299
Group II/ssDNA virus	Geminiviridae	Mastrevirus	Sugarcane streak Reunion virus	1.82E-3	21	Ori-C1-C4	Tip-date calibration under the Bayesian phylogenetic framework	(6)	485
Group II/ssDNA virus	Geminiviridae	Mastrevirus	Sugarcane streak Reunion virus	3.5E-4	32	Genome	Tip-date calibration under the Bayesian phylogenetic framework	(8)	515
Group II/ssDNA virus	Nanoviridae	Babuvirus	Banana bunchy top virus	1.43E-4	16	Genome	Internal-node calibration under the Bayesian phylogenetic framework	(9)	1000

Group II/ssDNA virus	Parvoviridae	Brevidensovirus	Infectious hypodermal and hematopoietic necrosis virus	1.39E-4	21	Capsid protein coding region	Tip-date calibration under the Bayesian phylogenetic framework	(10)	1000
Group II/ssDNA virus	Parvoviridae	Erythroparvovirus	Human B19	1.83E-4	28	Coding region	Tip-date calibration under the Bayesian phylogenetic framework	(11)	82
Group II/ssDNA virus	Parvoviridae	Erythroparvovirus	Human B19	1.72E-4	28	Coding region with unique gene rate; NS1	Tip-date calibration under the Bayesian phylogenetic framework	(11)	72
Group II/ssDNA virus	Parvoviridae	Erythroparvovirus	Human B19	1.74E-4	28	Coding region with unique gene rate; NS1	Tip-date calibration under the Bayesian phylogenetic framework	(11)	83
Group II/ssDNA virus	Parvoviridae	Erythroparvovirus	Human B19	2.07E-4	28	Coding region with unique gene rate; VP1	Tip-date calibration under the Bayesian phylogenetic framework	(11)	84
Group II/ssDNA virus	Parvoviridae	Erythroparvovirus	Human B19	2.04E-4	28	Coding region with unique gene rate; VP1	Tip-date calibration under the Bayesian phylogenetic framework	(11)	84
Group II/ssDNA virus	Parvoviridae	Erythroparvovirus	Human B19	1.90E-4	28	NS1 coding region	Tip-date calibration under the Bayesian phylogenetic framework	(11)	87
Group II/ssDNA virus	Parvoviridae	Erythroparvovirus	Human B19	1.14E-4	28	VP1	Tip-date calibration under the Bayesian phylogenetic framework	(11)	92
Group II/ssDNA virus	Parvoviridae	Erythroparvovirus	Human B19	2.60E-4	28	VP1 coding region	Tip-date calibration under the Bayesian phylogenetic framework	(11)	66
Group II/ssDNA virus	Parvoviridae	Erythroparvovirus	Human B19	1.20E-4	14	NS1 coding region	Tip-date calibration under the Bayesian phylogenetic framework	(12)	81
Group II/ssDNA virus	Parvoviridae	Erythroparvovirus	Human B19	2.00E-4	14	NS1-VP1	Tip-date calibration under the Bayesian phylogenetic framework	(12)	84
Group II/ssDNA virus	Parvoviridae	Erythroparvovirus	Human B19	2.30E-4	14	VP1 coding region	Tip-date calibration under the Bayesian phylogenetic framework	(12)	88
Group II/ssDNA virus	Parvoviridae	Erythroparvovirus	Human B19	3.64E-4	34	ORF2	Tip-date calibration under the Bayesian phylogenetic framework	(13)	97
Group II/ssDNA virus	Parvoviridae	Protoparvovirus	Canine parvovirus	7.9E-5	26	NS1 coding region	Tip-date calibration under the Bayesian phylogenetic framework	(14)	323
Group II/ssDNA virus	Parvoviridae	Protoparvovirus	Canine parvovirus	1.7E-4	24	VP2 coding region	Tip-date calibration under the Bayesian phylogenetic framework	(14)	366
Group II/ssDNA virus	Parvoviridae	Protoparvovirus	Canine parvovirus	1.7E-4	26	VP2 coding region	Tip-date calibration under the Bayesian phylogenetic framework	(14)	311
Group II/ssDNA virus	Parvoviridae	Protoparvovirus	Feline panleukopenia parvovirus	7.9E-5	28	NS1 coding region	Tip-date calibration under the Bayesian phylogenetic framework	(14)	469
Group II/ssDNA virus	Parvoviridae	Protoparvovirus	Feline panleukopenia parvovirus	9.4E-5	34	VP2 coding region	Tip-date calibration under the Bayesian phylogenetic framework	(14)	531
Group II/ssDNA virus	Parvoviridae	Protoparvovirus	Porcine parvovirus	5.39E-5	33	NS1 coding region	Tip-date calibration under the Bayesian phylogenetic framework	(15)	512
Group II/ssDNA virus	Parvoviridae	Protoparvovirus	Porcine parvovirus	3.02E-4	33	VP1 coding region	Tip-date calibration under the Bayesian phylogenetic framework	(15)	488
Group IV/(+)ssRNA virus	Arteriviridae	Arterivirus	Porcine reproductive and respiratory syndrome virus	3.29E-3	2	ORF5	Tip-date calibration under the Bayesian phylogenetic framework	(16)	495

Group IV/(+)ssRNA virus	Arteriviridae	Arterivirus	Porcine reproductive and respiratory syndrome virus	1.8E-3	3	ORF3,4, and 5	Tip-date calibration under the maximum-likelihood phylogenetic framework	(17)	505
Group IV/(+)ssRNA virus	Astroviridae	Mamastrovirus	Human astrovirus	1.03E-2	18	Outer-structural protein coding region	Tip-date calibration under the maximum-likelihood phylogenetic framework	(18)	1000
Group IV/(+)ssRNA virus	Caliciviridae	Lagovirus	Rabbit hemorrhagic disease virus	7.70E-04	53	Capsid protein coding region	Tip-date calibration under the Bayesian phylogenetic framework	(19)	1000
Group IV/(+)ssRNA virus	Caliciviridae	Norovirus	Norwalk virus	4.97E-3 (4.30E-3, 5.60E-3, 5.10E-3)	34	VP1 coding region	Tip-date calibration under the Bayesian phylogenetic framework	(20)	484
Group IV/(+)ssRNA virus	Caliciviridae	Norovirus	Norwalk virus	5.63E-3 (4.16E-3, 7.39E-3, 5.80E-3)	31	VP1 coding region	Tip-date calibration under the Bayesian phylogenetic framework	(21)	516
Group IV/(+)ssRNA virus	Coronaviridae	Alphacoronavirus	Human coronavirus 229E	3.85E-4 (3.28E-4, 6.17E-4, 2.82E-4)	33	S protein coding region	Tip-date calibration under the Bayesian phylogenetic framework	(22)	1000
Group IV/(+)ssRNA virus	Coronaviridae	Betacoronavirus	Bovine coronavirus	4.64E-4 (5.0E-4, 4.3E-4)	33	Spike protein coding region	Linear regression of the maximum-likelihood nucleotide substitution estimates against the years of isolation/ Tip-date calibration under the maximum-likelihood phylogenetic framework	(23)	1000
Group IV/(+)ssRNA virus	Coronaviridae	Betacoronavirus	SARS coronavirus	2.79E-3	2	ORF1	Tip-date calibration under the Bayesian phylogenetic framework	(24)	496
Group IV/(+)ssRNA virus	Coronaviridae	Betacoronavirus	SARS coronavirus	2.82E-3	4	ORF1	Tip-date calibration under the Bayesian phylogenetic framework	(25)	504
Group IV/(+)ssRNA virus	Coronaviridae	Gammacoronavirus	Avian coronavirus	9.40E-3	13	S1 coding region	Tip-date calibration under the Bayesian phylogenetic framework	(26)	336
Group IV/(+)ssRNA virus	Coronaviridae	Gammacoronavirus	Avian coronavirus	1.70E-3	25	S1 coding region	Tip-date calibration under the Bayesian phylogenetic framework	(26)	323
Group IV/(+)ssRNA virus	Coronaviridae	Gammacoronavirus	Avian coronavirus	2.40E-3	41	S1 coding region	Tip-date calibration under the Bayesian phylogenetic framework	(26)	341
Group IV/(+)ssRNA virus	Flaviviridae	Flavivirus	Dengue 1 virus	4.55E-4	54	E coding region	Tip-date calibration under the maximum-likelihood phylogenetic framework	(27)	499
Group IV/(+)ssRNA virus	Flaviviridae	Flavivirus	Dengue 1 virus	6.50E-4	43	E coding region	Tip-date calibration under the Bayesian phylogenetic framework	(28)	501
Group IV/(+)ssRNA virus	Flaviviridae	Flavivirus	Dengue 2 virus	6.07E-4	54	E coding region	Tip-date calibration under the maximum-likelihood phylogenetic framework	(27)	235
Group IV/(+)ssRNA virus	Flaviviridae	Flavivirus	Dengue 2 virus	5.66E-4	19	E coding region	Tip-date calibration under the maximum-likelihood phylogenetic framework	(29)	250
Group IV/(+)ssRNA virus	Flaviviridae	Flavivirus	Dengue 2 virus	6.50E-4	51	E coding region	Tip-date calibration under the Bayesian phylogenetic framework	(30)	250
Group IV/(+)ssRNA virus	Flaviviridae	Flavivirus	Dengue 2 virus	8.0E-4	21	E coding region	Tip-date calibration under the Bayesian phylogenetic framework	(31)	265
Group IV/(+)ssRNA virus	Flaviviridae	Flavivirus	Dengue 3 virus	9.01E-4	44	E coding region	Tip-date calibration under the maximum-likelihood phylogenetic framework	(27)	252
Group IV/(+)ssRNA virus	Flaviviridae	Flavivirus	Dengue 3 virus	1.03E-3	18	E coding region	Tip-date calibration under the Bayesian phylogenetic framework	(32)	244

Group IV/(+)ssRNA virus	Flaviviridae	Flavivirus	Dengue 3 virus	8.48E-4	7	E coding region	Tip-date calibration under the Bayesian phylogenetic framework	(33)	252
Group IV/(+)ssRNA virus	Flaviviridae	Flavivirus	Dengue 3 virus	8.9E-4	50	E coding region	Tip-date calibration under the Bayesian phylogenetic framework	(34)	252
Group IV/(+)ssRNA virus	Flaviviridae	Flavivirus	Dengue 4 virus	6.02E-4	38	E coding region	Tip-date calibration under the maximum-likelihood phylogenetic framework	(27)	338
Group IV/(+)ssRNA virus	Flaviviridae	Flavivirus	Dengue 4 virus	8.3E-4	18	E coding region	Tip-date calibration under the Bayesian phylogenetic framework	(31)	353
Group IV/(+)ssRNA virus	Flaviviridae	Flavivirus	Dengue 4 virus	1.07E-3	26	E coding region	Tip-date calibration under the Bayesian phylogenetic framework	(35)	309
Group IV/(+)ssRNA virus	Flaviviridae	Flavivirus	Japanese encephalitis virus	4.35E-04	74	Genome	Tip-date calibration under the Bayesian phylogenetic framework	(36)	1000
Group IV/(+)ssRNA virus	Flaviviridae	Flavivirus	Kyasanur forest disease virus	6.4E-4	49	Envelop-NS5	Tip-date calibration under the Bayesian phylogenetic framework	(37)	1000
Group IV/(+)ssRNA virus	Flaviviridae	Flavivirus	St. Louis encephalitis virus	2.17E-4	78	E coding region	Tip-date calibration under the Bayesian phylogenetic framework	(38)	503
Group IV/(+)ssRNA virus	Flaviviridae	Flavivirus	St. Louis encephalitis virus	4.1E-4	72	E coding region	Tip-date calibration under the Bayesian phylogenetic framework	(39)	497
Group IV/(+)ssRNA virus	Flaviviridae	Flavivirus	West Nile virus	8.5E-4	8	E coding region	Tip-date calibration under the Bayesian phylogenetic framework	(40)	1000
Group IV/(+)ssRNA virus	Flaviviridae	Flavivirus	Yellow fever virus	4.2E-4	76	prM/E	Tip-date calibration under the Bayesian phylogenetic framework	(41)	1000
Group IV/(+)ssRNA virus	Flaviviridae	Hepacivirus	Hepatitis C virus	2.1E-3	17	E2-P7-NS2	Tip-date calibration under the Bayesian phylogenetic framework	(42)	100
Group IV/(+)ssRNA virus	Flaviviridae	Hepacivirus	Hepatitis C virus	1.3E-3	30	E2-P7-NS2	Tip-date calibration under the Bayesian phylogenetic framework	(42)	118
Group IV/(+)ssRNA virus	Flaviviridae	Hepacivirus	Hepatitis C virus	1.2E-3	17	NSSB coding region	Tip-date calibration under the Bayesian phylogenetic framework	(42)	111
Group IV/(+)ssRNA virus	Flaviviridae	Hepacivirus	Hepatitis C virus	1.0E-3	30	NSSB coding region	Tip-date calibration under the Bayesian phylogenetic framework	(42)	107
Group IV/(+)ssRNA virus	Flaviviridae	Hepacivirus	Hepatitis C virus	1.9E-3	17	NSSB coding region with E2-P7-NS2 rate prior	Tip-date calibration under the Bayesian phylogenetic framework	(42)	105
Group IV/(+)ssRNA virus	Flaviviridae	Hepacivirus	Hepatitis C virus	1.0E-3	30	NSSB with E2-P7-NS2 rate prior	Tip-date calibration under the Bayesian phylogenetic framework	(42)	134
Group IV/(+)ssRNA virus	Flaviviridae	Hepacivirus	Hepatitis C virus	1.12E-3 (1.12E-3, 1.11E-3, 1.13E-3, 1.11E-3)	19	Genome	Tip-date calibration under the Bayesian phylogenetic framework	(43)	127
Group IV/(+)ssRNA virus	Flaviviridae	Hepacivirus	Hepatitis C virus	1.17E-3 (1.04E-3, 1.25E-3, 1.24E-3, 1.18E-3)	19	Genome	Tip-date calibration under the Bayesian phylogenetic framework	(43)	97
Group IV/(+)ssRNA virus	Flaviviridae	Hepacivirus	Hepatitis C virus	1.42E-3 (1.30E-3, 1.44E-3, 1.47E-3, 1.48E-3)	19	Genome	Tip-date calibration under the Bayesian phylogenetic framework	(43)	101

Group IV/(+)ssRNA virus	Flaviviridae	Pegivirus	GB virus C	3.42E-3 (4.13E-3, 4.15E-3, 2.10E-3, 4.45E-3, 4.13E-3, 2.09E-3, 5.03E-3, 4.99E-3, 1.85E-3)	9	5'UTR	Tip-date calibration under the Bayesian phylogenetic framework	(44)	267
Group IV/(+)ssRNA virus	Flaviviridae	Pegivirus	GB virus C	3.50E-2 (5.43E-2, 4.33E-2, 3.28E-2, 1.94E-2, 3.95E-2, 3.95E-2, 2.49E-2, 4.30E-2, 3.16E-2)	4	E1 coding region	Tip-date calibration under the Bayesian phylogenetic framework	(44)	243
Group IV/(+)ssRNA virus	Flaviviridae	Pegivirus	GB virus C	9.33E-3 (2.19E-2, 1.46E-2, 3.53E-3, 2.74E-2, 7.92E-3, 2.61E-3, 2.89E-2, 8.67E-3, 3.34E-3)	3	E2 coding region	Tip-date calibration under the Bayesian phylogenetic framework	(44)	260
Group IV/(+)ssRNA virus	Flaviviridae	Pegivirus	GB virus C	1.65E-2 (3.55E-2, 2.12E-2, 1.52E-2, 3.58E-2, 8.49E-3, 6.99E-3, 3.24E-2, 1.14E-2, 1.02E-2)	4	NS5b coding region	Tip-date calibration under the Bayesian phylogenetic framework	(44)	230
Group IV/(+)ssRNA virus	Luteoviridae	Luteovirus	Barley yellow dwarf virus	1.5E-3	89	CP coding region	Tip-date calibration under the Bayesian phylogenetic framework	(45)	232
Group IV/(+)ssRNA virus	Luteoviridae	Luteovirus	Barley yellow dwarf virus	6.7E-3	89	RdRp coding region	Tip-date calibration under the Bayesian phylogenetic framework	(45)	262
Group IV/(+)ssRNA virus	Luteoviridae	Luteovirus	Barley yellow dwarf virus	6.0E-4	89	RTD	Tip-date calibration under the Bayesian phylogenetic framework	(45)	240
Group IV/(+)ssRNA virus	Luteoviridae	Luteovirus	Barley yellow dwarf virus	3.16E-4	2	Genome	Tip-date calibration under the Bayesian phylogenetic framework	(46)	266
Group IV/(+)ssRNA virus	Luteoviridae	Luteovirus	Soybean dwarf virus	6.0E-4	17	CP coding region	Tip-date calibration under the Bayesian phylogenetic framework	(45)	514
Group IV/(+)ssRNA virus	Luteoviridae	Luteovirus	Soybean dwarf virus	2.3E-3	17	RTD	Tip-date calibration under the Bayesian phylogenetic framework	(45)	486
Group IV/(+)ssRNA virus	Luteoviridae	Polerovirus	Beet chlorosis virus	2.7E-3	20	CP coding region	Tip-date calibration under the Bayesian phylogenetic framework	(45)	334
Group IV/(+)ssRNA virus	Luteoviridae	Polerovirus	Beet chlorosis virus	7.2E-3	20	P0 coding region	Tip-date calibration under the Bayesian phylogenetic framework	(45)	332
Group IV/(+)ssRNA virus	Luteoviridae	Polerovirus	Beet chlorosis virus	1.6E-3	20	RdRp coding region	Tip-date calibration under the Bayesian phylogenetic framework	(45)	334
Group IV/(+)ssRNA virus	Luteoviridae	Polerovirus	Beet mild yellowing virus	7.7E-4	25	P0 coding region	Tip-date calibration under the Bayesian phylogenetic framework	(45)	508
Group IV/(+)ssRNA virus	Luteoviridae	Polerovirus	Beet mild yellowing virus	3.7E-4	25	RdRp coding region	Tip-date calibration under the Bayesian phylogenetic framework	(45)	492
Group IV/(+)ssRNA virus	Luteoviridae	Polerovirus	Cereal yellow dwarf virus-RPV	7.4E-4	81	CP coding region	Tip-date calibration under the Bayesian phylogenetic framework	(45)	363
Group IV/(+)ssRNA virus	Luteoviridae	Polerovirus	Cereal yellow dwarf virus-RPV	2.3E-2	81	RdRp coding region	Tip-date calibration under the Bayesian phylogenetic framework	(45)	316

Group IV/(+)ssRNA virus	Luteoviridae	Polerovirus	Cereal yellow dwarf virus-RPV	3.3E-3	81	RTD	Tip-date calibration under the Bayesian phylogenetic framework	(45)	321
Group IV/(+)ssRNA virus	Luteoviridae	Polerovirus	Cucurbit aphid-borne yellows virus	3.5E-2	5	CP coding region	Tip-date calibration under the Bayesian phylogenetic framework	(45)	503
Group IV/(+)ssRNA virus	Luteoviridae	Polerovirus	Cucurbit aphid-borne yellows virus	4.3E-3	5	RdRp coding region	Tip-date calibration under the Bayesian phylogenetic framework	(45)	497
Group IV/(+)ssRNA virus	Luteoviridae	Polerovirus	Potato leafroll virus	6.2E-4	34	CP coding region	Tip-date calibration under the Bayesian phylogenetic framework	(45)	488
Group IV/(+)ssRNA virus	Luteoviridae	Polerovirus	Potato leafroll virus	3.4E-4	34	P0 coding region	Tip-date calibration under the Bayesian phylogenetic framework	(45)	512
Group IV/(+)ssRNA virus	Luteoviridae	Polerovirus	Sugarcane yellow leaf virus	1.1E-3	19	CP coding region	Tip-date calibration under the Bayesian phylogenetic framework	(45)	311
Group IV/(+)ssRNA virus	Luteoviridae	Polerovirus	Sugarcane yellow leaf virus	1.2E-3	19	P0 coding region	Tip-date calibration under the Bayesian phylogenetic framework	(45)	351
Group IV/(+)ssRNA virus	Luteoviridae	Polerovirus	Sugarcane yellow leaf virus	1.4E-4	19	RdRp coding region	Tip-date calibration under the Bayesian phylogenetic framework	(45)	338
Group IV/(+)ssRNA virus	Luteoviridae	Polerovirus	Turnip yellows virus	8.6E-4	26	CP coding region	Tip-date calibration under the Bayesian phylogenetic framework	(45)	317
Group IV/(+)ssRNA virus	Luteoviridae	Polerovirus	Turnip yellows virus	2.7E-3	26	P0 coding region	Tip-date calibration under the Bayesian phylogenetic framework	(45)	350
Group IV/(+)ssRNA virus	Luteoviridae	Polerovirus	Turnip yellows virus	1.0E-3	26	RdRp coding region	Tip-date calibration under the Bayesian phylogenetic framework	(45)	333
Group IV/(+)ssRNA virus	Picornaviridae	Aphthovirus	Foot-and-mouth disease virus	1.45E-3	38	3DPol coding region	Tip-date calibration under the Bayesian phylogenetic framework	(47)	341
Group IV/(+)ssRNA virus	Picornaviridae	Aphthovirus	Foot-and-mouth disease virus	1.46E-3	75	Genome	Tip-date calibration under the Bayesian phylogenetic framework	(48)	321
Group IV/(+)ssRNA virus	Picornaviridae	Aphthovirus	Foot-and-mouth disease virus	2.26E-5	0.58	Genome	Tip-date calibration under the Bayesian phylogenetic framework	(49)	338
Group IV/(+)ssRNA virus	Picornaviridae	Cardiovirus	Encephalomyocarditis virus	1.61E-3	22	VP1 coding region	Tip-date calibration under the Bayesian phylogenetic framework	(47)	1000
Group IV/(+)ssRNA virus	Picornaviridae	Enterovirus	Enterovirus A	5.53E-3	24	3DPol coding region	Tip-date calibration under the Bayesian phylogenetic framework	(47)	252
Group IV/(+)ssRNA virus	Picornaviridae	Enterovirus	Enterovirus A	3.66E-3	29	1D coding region	Tip-date calibration under the Bayesian phylogenetic framework	(50)	246
Group IV/(+)ssRNA virus	Picornaviridae	Enterovirus	Enterovirus A	3.19E-3	40	3CD coding region	Tip-date calibration under the Bayesian phylogenetic framework	(50)	261
Group IV/(+)ssRNA virus	Picornaviridae	Enterovirus	Enterovirus A	8.65E-3	59	VP1 coding region	Tip-date calibration under the Bayesian phylogenetic framework	(51)	241
Group IV/(+)ssRNA virus	Picornaviridae	Enterovirus	Enterovirus B	5.73E-3	48	VP1 coding region	Tip-date calibration under the Bayesian phylogenetic framework	(47)	194
Group IV/(+)ssRNA virus	Picornaviridae	Enterovirus	Enterovirus B	5.27E-3	62	VP1 coding region	Tip-date calibration under the Bayesian phylogenetic framework	(47)	223
Group IV/(+)ssRNA virus	Picornaviridae	Enterovirus	Enterovirus B	4.2E-3	26	VP1-P1	Tip-date calibration under the Bayesian phylogenetic framework	(52)	180
Group IV/(+)ssRNA virus	Picornaviridae	Enterovirus	Enterovirus B	8.8E-3	9	VP1 coding region	Tip-date calibration under the Bayesian phylogenetic framework	(53)	193

Group IV/(+)ssRNA virus	Picornaviridae	Enterovirus	Enterovirus B	8.5E-3	49	VP1 coding region	Tip-date calibration under the Bayesian phylogenetic framework	(53)	210
Group IV/(+)ssRNA virus	Picornaviridae	Enterovirus	Enterovirus C	11.68E-3	24	3DPol coding region	Tip-date calibration under the Bayesian phylogenetic framework	(47)	521
Group IV/(+)ssRNA virus	Picornaviridae	Enterovirus	Enterovirus C	9E-3	10	VP1-2A	Linear regression of neighbour-joining nucleotide substitution estimates against the years of isolation	(54)	479
Group IV/(+)ssRNA virus	Picornaviridae	Hepatovirus	Hepatitis A virus	8.9E-4	32	3DPol coding region	Tip-date calibration under the Bayesian phylogenetic framework	(47)	182
Group IV/(+)ssRNA virus	Picornaviridae	Hepatovirus	Hepatitis A virus	1.73E-4	33	Genome	Tip-date calibration under the Bayesian phylogenetic framework	(55)	219
Group IV/(+)ssRNA virus	Picornaviridae	Hepatovirus	Hepatitis A virus	1.99E-4	33	Genome	Tip-date calibration under the Bayesian phylogenetic framework	(55)	206
Group IV/(+)ssRNA virus	Picornaviridae	Hepatovirus	Hepatitis A virus	2.38E-4	17	VP1 (3rd codon)	Tip-date calibration under the Bayesian phylogenetic framework	(56)	204
Group IV/(+)ssRNA virus	Picornaviridae	Hepatovirus	Hepatitis A virus	9.76E-4	17	VP1 coding region	Tip-date calibration under the Bayesian phylogenetic framework	(56)	189
Group IV/(+)ssRNA virus	Picornaviridae	Parechovirus	Human parechovirus	2.96E-3	34	3DPol coding region	Tip-date calibration under the Bayesian phylogenetic framework	(47)	311
Group IV/(+)ssRNA virus	Picornaviridae	Parechovirus	Human parechovirus	2.21E-3	51	P1 coding region	Tip-date calibration under the Bayesian phylogenetic framework	(57)	352
Group IV/(+)ssRNA virus	Picornaviridae	Parechovirus	Human parechovirus	2.79E-3	32	VP1 coding region	Tip-date calibration under the Bayesian phylogenetic framework	(57)	337
Group IV/(+)ssRNA virus	Picornaviridae	Teschovirus	Porcine teschovirus	1.62E-3	50	VP1 coding region	Tip-date calibration under the Bayesian phylogenetic framework	(47)	1000
Group IV/(+)ssRNA virus	Potyviridae	Potyvirus	American cowpea aphid-borne mosaic virus	1.397E-4	500	cCP coding region	Dividing an average maximum-likelihood nucleotide substitution estimate by the timescale of evolution, inferred from human migration history	(58)	1000
Group IV/(+)ssRNA virus	Potyviridae	Potyvirus	American papaya ringspot virus	1.25E-4	300	cCP coding region	Dividing an average maximum-likelihood nucleotide substitution estimate by the timescale of evolution, inferred from human migration history	(58)	1000
Group IV/(+)ssRNA virus	Potyviridae	Potyvirus	Australian potyvirus	1.173E-4	75	cCP coding region	Dividing an average maximum-likelihood nucleotide substitution estimate by the timescale of evolution, inferred from human migration history	(58)	1000
Group IV/(+)ssRNA virus	Potyviridae	Potyvirus	Plum poxvirus	1.40E-4	100	cCP coding region	Dividing an average maximum-likelihood nucleotide substitution estimate by the timescale of evolution, inferred from the date of the Plum pox disease outbreak in Bulgaria	(58)	1000
Group IV/(+)ssRNA virus	Potyviridae	Potyvirus	Zucchini yellow mosaic virus	5.0E-4	22	CP coding region	Tip-date calibration under the Bayesian phylogenetic framework	(59)	1000
Group IV/(+)ssRNA virus	Togaviridae	Alphavirus	Fort Morgan virus	1.81E-4	9	Capsid-E3-E2-6K-E1	Tip-date calibration under the Bayesian phylogenetic framework	(60)	499

Group IV/(+)ssRNA virus	Togaviridae	Alphavirus	Fort Morgan virus	2.64E-4	32	Capsid-E3-E2-6K-E1	Tip-date calibration under the Bayesian phylogenetic framework	(60)	501
Group IV/(+)ssRNA virus	Togaviridae	Alphavirus	Ross River virus	7.53E-4 (8.13E-4, 8.55E-4, 6.91E-4, 7.45E-4, 6.78E-4)	45	E2 coding region	Tip-date calibration under the Bayesian phylogenetic framework	(61)	1000
Group IV/(+)ssRNA virus	Togaviridae	Alphavirus	Venezuelan equine encephalitis virus	1.28E-4	54	Structural polyprotein coding region	Tip-date calibration under the Bayesian phylogenetic framework	(62)	1000
Group IV/(+)ssRNA virus	Unclassified	Sobemovirus	Rice Yellow Mottle Virus	11.7E-4	40	CP coding region	Tip-date calibration under the Bayesian phylogenetic framework	(63)	346
Group IV/(+)ssRNA virus	Unclassified	Sobemovirus	Rice Yellow Mottle Virus	7.3E-4	40	CP coding region	Tip-date calibration under the Bayesian phylogenetic framework	(64)	325
Group IV/(+)ssRNA virus	Unclassified	Sobemovirus	Rice Yellow Mottle Virus	7.7E-4	40	CP coding region	Tip-date calibration under the Bayesian phylogenetic framework	(64)	329
Group IV/(+)ssRNA virus	Virgaviridae	Tobamovirus	Cucumber green mottle mosaic virus (nested in tobamovirus)	8.0E-4	38	CP coding region	Tip-date calibration under the Bayesian phylogenetic framework	(65)	0*
Group IV/(+)ssRNA virus	Virgaviridae	Tobamovirus	Cucumber green mottle mosaic virus (nested in tobamovirus)	6.7E-4	38	MP coding region	Tip-date calibration under the Bayesian phylogenetic framework	(65)	0*
Group IV/(+)ssRNA virus	Virgaviridae	Tobamovirus	Cucumber green mottle mosaic virus (nested in tobamovirus)	8.0E-5	38	RdRp coding region	Tip-date calibration under the Bayesian phylogenetic framework	(65)	0*
Group IV/(+)ssRNA virus	Virgaviridae	Tobamovirus	Odontoglossum ringspot virus (nested in tobamovirus)	7.8E-4	44	CP coding region	Tip-date calibration under the Bayesian phylogenetic framework	(65)	0*
Group IV/(+)ssRNA virus	Virgaviridae	Tobamovirus	Odontoglossum ringspot virus (nested in tobamovirus)	1.3E-3	44	MP coding region	Tip-date calibration under the Bayesian phylogenetic framework	(65)	0*
Group IV/(+)ssRNA virus	Virgaviridae	Tobamovirus	Odontoglossum ringspot virus (nested in tobamovirus)	7.7E-4	44	RdRp coding region	Tip-date calibration under the Bayesian phylogenetic framework	(65)	0*
Group IV/(+)ssRNA virus	Virgaviridae	Tobamovirus	Odontoglossum ringspot virus (nested in tobamovirus)	8.8E-4	44	RdRp coding region	Tip-date calibration under the Bayesian phylogenetic framework	(65)	0*
Group IV/(+)ssRNA virus	Virgaviridae	Tobamovirus	Pepper mild mottle virus (nested in tobamovirus)	1.8E-4	36	CP coding region	Tip-date calibration under the Bayesian phylogenetic framework	(65)	0*
Group IV/(+)ssRNA virus	Virgaviridae	Tobamovirus	Pepper mild mottle virus (nested in tobamovirus)	8.9E-4	36	MP coding region	Tip-date calibration under the Bayesian phylogenetic framework	(65)	0*
Group IV/(+)ssRNA virus	Virgaviridae	Tobamovirus	Pepper mild mottle virus (nested in tobamovirus)	2.6E-4	36	RdRp coding region	Tip-date calibration under the Bayesian phylogenetic framework	(65)	0*
Group IV/(+)ssRNA virus	Virgaviridae	Tobamovirus	Pepper mild mottle virus (nested in tobamovirus)	2.6E-4	36	RdRp coding region	Tip-date calibration under the Bayesian phylogenetic framework	(65)	0*
Group IV/(+)ssRNA virus	Virgaviridae	Tobamovirus	Tobacco mild green mosaic virus (nested in tobamovirus)	1.7E-4	101	CP coding region	Tip-date calibration under the Bayesian phylogenetic framework	(65)	0*
Group IV/(+)ssRNA virus	Virgaviridae	Tobamovirus	Tobacco mild green mosaic virus (nested in tobamovirus)	1.3E-4	101	RdRp coding region	Tip-date calibration under the Bayesian phylogenetic framework	(65)	0*
Group IV/(+)ssRNA virus	Virgaviridae	Tobamovirus	Tobacco mild green mosaic virus (nested in tobamovirus)	1.4E-4	101	RdRp coding region	Tip-date calibration under the Bayesian phylogenetic framework	(65)	0*
Group IV/(+)ssRNA virus	Virgaviridae	Tobamovirus	Tobacco mosaic virus (nested in tobamovirus)	1.6E-4	109	CP coding region	Tip-date calibration under the Bayesian phylogenetic framework	(65)	0*
Group IV/(+)ssRNA virus	Virgaviridae	Tobamovirus	Tobacco mosaic virus (nested in tobamovirus)	2.9E-4	109	RdRp coding region	Tip-date calibration under the Bayesian phylogenetic framework	(65)	0*

Group IV/(+)ssRNA virus	Virgaviridae	Tobamovirus	Tobacco mosaic virus (nested in tobamovirus)	7.9E-4	109	RdRp coding region	Tip-date calibration under the Bayesian phylogenetic framework	(65)	0*
Group V/(-)ssRNA virus	Bunyaviridae	Hantavirus	Araraquara hantavirus (nested in rodent hantavirus)	5.11E-3 (8.65E-3, 9.05E-3, 1.08E-2, 2.68E-3, 2.62E-3, 3.01E-3)	6	G1 coding region	Tip-date calibration under the Bayesian phylogenetic framework	(66)	248
Group V/(-)ssRNA virus	Bunyaviridae	Hantavirus	Araraquara hantavirus (nested in rodent hantavirus)	3.34E-3 (2.67E-3, 2.52E-3, 6.26E-3, 3.01E-3, 2.98E-3, 3.69E-3)	6	G2 coding region	Tip-date calibration under the Bayesian phylogenetic framework	(66)	247
Group V/(-)ssRNA virus	Bunyaviridae	Hantavirus	Araraquara hantavirus (nested in rodent hantavirus)	2.67E-3 (2.49E-3, 2.43E-3 3.23E-3, 2.48E-3, 2.63E-3, 2.84E-3)	6	N coding region	Tip-date calibration under the Bayesian phylogenetic framework	(66)	246
Group V/(-)ssRNA virus	Bunyaviridae	Hantavirus	Dobrava hantavirus (nested in rodent hantavirus)	3.23E-4 (2.99E-4, 2.80E-4, 4.74E-4, 2.66E-4, 2.90E-4, 3.74E-4)	21	N coding region	Tip-date calibration under the Bayesian phylogenetic framework	(66)	741
Group V/(-)ssRNA virus	Bunyaviridae	Hantavirus	Puumala hantavirus (nested in Puumala and Tula hantavirus, and rodent hantavirus)	5.75E-4 (6.09E-4, 5.41E-4, 6.22E-4, 5.20E-4, 5.51E-4, 6.14E-4)	25	N coding region	Tip-date calibration under the Bayesian phylogenetic framework	(66)	0*
Group V/(-)ssRNA virus	Bunyaviridae	Hantavirus	Rodent hantavirus	6.76E-4	28	S segment	Tip-date calibration under the Bayesian phylogenetic framework	(67)	0*
Group V/(-)ssRNA virus	Bunyaviridae	Hantavirus	Sin Nombre virus (nested in rodent hantavirus)	6.76E-3	12	M segment	Tip-date calibration under the Bayesian phylogenetic framework	(68)	362
Group V/(-)ssRNA virus	Bunyaviridae	Hantavirus	Sin Nombre virus (nested in rodent hantavirus)	1.93E-3	12	S segment	Tip-date calibration under the Bayesian phylogenetic framework	(68)	379
Group V/(-)ssRNA virus	Bunyaviridae	Hantavirus	Tula hantavirus (nested in Puumala and Tula hantavirus, and rodent hantavirus)	1.25E-2 (1.99E-2, 2.10E-2, 1.84E-2, 8.07E-3, 6.77E-3, 8.87E-3)	9	N coding region	Tip-date calibration under the Bayesian phylogenetic framework	(66)	252
Group V/(-)ssRNA virus	Bunyaviridae	Nairovirus	Crimean-Congo hemorrhagic fever virus	1.01E-4	47	L segment	Tip-date calibration under the maximum-likelihood phylogenetic framework	(69)	123
Group V/(-)ssRNA virus	Bunyaviridae	Nairovirus	Crimean-Congo hemorrhagic fever virus	1.02E-4	47	L segment	Tip-date calibration under the maximum-likelihood phylogenetic framework	(69)	96
Group V/(-)ssRNA virus	Bunyaviridae	Nairovirus	Crimean-Congo hemorrhagic fever virus	0.92E-4	47	M segment	Tip-date calibration under the maximum-likelihood phylogenetic framework	(69)	118
Group V/(-)ssRNA virus	Bunyaviridae	Nairovirus	Crimean-Congo hemorrhagic fever virus	1.22E-4	47	M segment	Tip-date calibration under the maximum-likelihood phylogenetic framework	(69)	99
Group V/(-)ssRNA virus	Bunyaviridae	Nairovirus	Crimean-Congo hemorrhagic fever virus	0.27E-4	47	S segment	Tip-date calibration under the maximum-likelihood phylogenetic framework	(69)	105
Group V/(-)ssRNA virus	Bunyaviridae	Nairovirus	Crimean-Congo hemorrhagic fever virus	0.34E-4	47	S segment	Tip-date calibration under the maximum-likelihood phylogenetic framework	(69)	111
Group V/(-)ssRNA virus	Bunyaviridae	Nairovirus	Crimean-Congo hemorrhagic fever virus	5.8E-5	47	L segment	Tip-date calibration under the Bayesian phylogenetic framework	(70)	114

Group V/(-)ssRNA virus	Bunyaviridae	Nairovirus	Crimean-Congo hemorrhagic fever virus	1.52E-4	47	M segment	Tip-date calibration under the Bayesian phylogenetic framework	(70)	124
Group V/(-)ssRNA virus	Bunyaviridae	Nairovirus	Crimean-Congo hemorrhagic fever virus	1.09E-4	49	S segment	Tip-date calibration under the Bayesian phylogenetic framework	(70)	110
Group V/(-)ssRNA virus	Bunyaviridae	Phlebovirus	Rift valley fever virus	2.78E-4	56	L segment	Tip-date calibration under the Bayesian phylogenetic framework	(71)	175
Group V/(-)ssRNA virus	Bunyaviridae	Phlebovirus	Rift valley fever virus	2.42E-4	56	M segment	Tip-date calibration under the Bayesian phylogenetic framework	(71)	166
Group V/(-)ssRNA virus	Bunyaviridae	Phlebovirus	Rift valley fever virus	2.35E-4	56	S segment	Tip-date calibration under the Bayesian phylogenetic framework	(71)	152
Group V/(-)ssRNA virus	Bunyaviridae	Phlebovirus	Rift valley fever virus	2.8E-4	0.5	L segment	Tip-date calibration under the Bayesian phylogenetic framework	(72)	167
Group V/(-)ssRNA virus	Bunyaviridae	Phlebovirus	Rift valley fever virus	3.6E-4	0.5	M segment	Tip-date calibration under the Bayesian phylogenetic framework	(72)	175
Group V/(-)ssRNA virus	Bunyaviridae	Phlebovirus	Rift valley fever virus	3.9E-4	0.5	S segment	Tip-date calibration under the Bayesian phylogenetic framework	(72)	165
Group V/(-)ssRNA virus	Orthomyxoviridae	Influenzavirus A	Influenza A virus	3.67E-3	0.25	HA coding region	Tip-date calibration under the Bayesian phylogenetic framework	(73)	31
Group V/(-)ssRNA virus	Orthomyxoviridae	Influenzavirus A	Influenza A virus	2.55E-3	0.25	MP coding region	Tip-date calibration under the Bayesian phylogenetic framework	(73)	35
Group V/(-)ssRNA virus	Orthomyxoviridae	Influenzavirus A	Influenza A virus	3.65E-3	0.25	NA coding region	Tip-date calibration under the Bayesian phylogenetic framework	(73)	35
Group V/(-)ssRNA virus	Orthomyxoviridae	Influenzavirus A	Influenza A virus	2.59E-3	0.25	NP coding region	Tip-date calibration under the Bayesian phylogenetic framework	(73)	29
Group V/(-)ssRNA virus	Orthomyxoviridae	Influenzavirus A	Influenza A virus	2.62E-3	0.25	NS coding region	Tip-date calibration under the Bayesian phylogenetic framework	(73)	24
Group V/(-)ssRNA virus	Orthomyxoviridae	Influenzavirus A	Influenza A virus	2.45E-3	0.25	PA coding region	Tip-date calibration under the Bayesian phylogenetic framework	(73)	24
Group V/(-)ssRNA virus	Orthomyxoviridae	Influenzavirus A	Influenza A virus	2.34E-3	0.25	PB1 coding region	Tip-date calibration under the Bayesian phylogenetic framework	(73)	26
Group V/(-)ssRNA virus	Orthomyxoviridae	Influenzavirus A	Influenza A virus	2.60E-3	0.25	PB2 coding region	Tip-date calibration under the Bayesian phylogenetic framework	(73)	30
Group V/(-)ssRNA virus	Orthomyxoviridae	Influenzavirus A	Influenza A virus	3.92E-3	29	HA coding region	Tip-date calibration under the Bayesian phylogenetic framework	(74)	38
Group V/(-)ssRNA virus	Orthomyxoviridae	Influenzavirus A	Influenza A virus	2.49E-3	32	MP1 coding region	Tip-date calibration under the Bayesian phylogenetic framework	(74)	28
Group V/(-)ssRNA virus	Orthomyxoviridae	Influenzavirus A	Influenza A virus	3.61E-3	48	NA coding region	Tip-date calibration under the Bayesian phylogenetic framework	(74)	42
Group V/(-)ssRNA virus	Orthomyxoviridae	Influenzavirus A	Influenza A virus	3.17E-3	42	NP coding region	Tip-date calibration under the Bayesian phylogenetic framework	(74)	28
Group V/(-)ssRNA virus	Orthomyxoviridae	Influenzavirus A	Influenza A virus	3.87E-3	42	NS1 coding region	Tip-date calibration under the Bayesian phylogenetic framework	(74)	30
Group V/(-)ssRNA virus	Orthomyxoviridae	Influenzavirus A	Influenza A virus	3.48E-3	49	PA coding region	Tip-date calibration under the Bayesian phylogenetic framework	(74)	26
Group V/(-)ssRNA virus	Orthomyxoviridae	Influenzavirus A	Influenza A virus	2.86E-3	42	PB1 coding region	Tip-date calibration under the Bayesian phylogenetic framework	(74)	22

Group V/(-)ssRNA virus	Orthomyxoviridae	Influenzavirus A	Influenza A virus	3.15E-3	49	PB2 coding region	Tip-date calibration under the Bayesian phylogenetic framework	(74)	41
Group V/(-)ssRNA virus	Orthomyxoviridae	Influenzavirus A	Influenza A virus	5.42E-4	37	M segment	Linear regression of neighbour-joining nucleotide substitution estimates against the years of isolation	(75)	30
Group V/(-)ssRNA virus	Orthomyxoviridae	Influenzavirus A	Influenza A virus	5.06E-4	37	NS segment	Linear regression of neighbour-joining nucleotide substitution estimates against the years of isolation	(75)	33
Group V/(-)ssRNA virus	Orthomyxoviridae	Influenzavirus A	Influenza A virus	7.84E-3	1	NA coding region	Tip-date calibration under the Bayesian phylogenetic framework	(76)	30
Group V/(-)ssRNA virus	Orthomyxoviridae	Influenzavirus A	Influenza A virus	4.31E-3	11	NA coding region	Tip-date calibration under the Bayesian phylogenetic framework	(77)	21
Group V/(-)ssRNA virus	Orthomyxoviridae	Influenzavirus A	Influenza A virus	1.82E-3	23	NA coding region	Tip-date calibration under the Bayesian phylogenetic framework	(77)	19
Group V/(-)ssRNA virus	Orthomyxoviridae	Influenzavirus A	Influenza A virus	3.12E-3	29	NA coding region	Tip-date calibration under the Bayesian phylogenetic framework	(77)	29
Group V/(-)ssRNA virus	Orthomyxoviridae	Influenzavirus A	Influenza A virus	2.11E-3	39	NA coding region	Tip-date calibration under the Bayesian phylogenetic framework	(77)	32
Group V/(-)ssRNA virus	Orthomyxoviridae	Influenzavirus A	Influenza A virus	3.20E-3	72	NA coding region	Tip-date calibration under the Bayesian phylogenetic framework	(77)	20
Group V/(-)ssRNA virus	Orthomyxoviridae	Influenzavirus A	Influenza A virus	3.28E-3	76	NA coding region	Tip-date calibration under the Bayesian phylogenetic framework	(77)	27
Group V/(-)ssRNA virus	Orthomyxoviridae	Influenzavirus A	Influenza A virus	3.43E-3	1	NP coding region	Tip-date calibration under the Bayesian phylogenetic framework	(78)	31
Group V/(-)ssRNA virus	Orthomyxoviridae	Influenzavirus A	Influenza A virus	2.8E-3	30	NP coding region	Tip-date calibration under the Bayesian phylogenetic framework	(78)	38
Group V/(-)ssRNA virus	Orthomyxoviridae	Influenzavirus A	Influenza A virus	9E-4	45	NP coding region	Tip-date calibration under the Bayesian phylogenetic framework	(78)	27
Group V/(-)ssRNA virus	Orthomyxoviridae	Influenzavirus A	Influenza A virus	2.25E-3	55	NP coding region	Tip-date calibration under the Bayesian phylogenetic framework	(78)	35
Group V/(-)ssRNA virus	Orthomyxoviridae	Influenzavirus A	Influenza A virus	2.29E-3	79	NP coding region	Tip-date calibration under the Bayesian phylogenetic framework	(78)	15
Group V/(-)ssRNA virus	Orthomyxoviridae	Influenzavirus A	Influenza A virus	3.41E-3	82	NP coding region	Tip-date calibration under the Bayesian phylogenetic framework	(78)	26
Group V/(-)ssRNA virus	Orthomyxoviridae	Influenzavirus A	Influenza A virus	1.98E-3	91	NP coding region	Tip-date calibration under the Bayesian phylogenetic framework	(78)	28
Group V/(-)ssRNA virus	Orthomyxoviridae	Influenzavirus A	Influenza A virus	2.28E-3	27	NA coding region	Linear regression of the maximum-likelihood nucleotide substitution estimates against the years of isolation	(79)	27
Group V/(-)ssRNA virus	Orthomyxoviridae	Influenzavirus A	Influenza A virus	2.58E-3	27	NA coding region	Linear regression of the maximum-likelihood nucleotide substitution estimates against the years of isolation	(79)	43
Group V/(-)ssRNA virus	Orthomyxoviridae	Influenzavirus B	Influenza B virus	1.39E-3	13	HA coding region	Linear regression of neighbour-joining nucleotide substitution estimates against the years of isolation	(80)	132

Group V/(-)ssRNA virus	Orthomyxoviridae	Influenzavirus B	Influenza B virus	2.41E-3	19	HA coding region	Linear regression of neighbour-joining nucleotide substitution estimates against the years of isolation	(80)	145
Group V/(-)ssRNA virus	Orthomyxoviridae	Influenzavirus B	Influenza B virus	1.09E-3	19	M coding region	Linear regression of neighbour-joining nucleotide substitution estimates against the years of isolation	(80)	128
Group V/(-)ssRNA virus	Orthomyxoviridae	Influenzavirus B	Influenza B virus	1.31E-3	9	M segment	Linear regression of neighbour-joining nucleotide substitution estimates against the years of isolation	(80)	147
Group V/(-)ssRNA virus	Orthomyxoviridae	Influenzavirus B	Influenza B virus	0.95E-3	19	NP coding region	Linear regression of neighbour-joining nucleotide substitution estimates against the years of isolation	(80)	151
Group V/(-)ssRNA virus	Orthomyxoviridae	Influenzavirus B	Influenza B virus	0.45E-3	14	NS coding region	Linear regression of neighbour-joining nucleotide substitution estimates against the years of isolation	(80)	158
Group V/(-)ssRNA virus	Orthomyxoviridae	Influenzavirus B	Influenza B virus	0.87E-3	21	NS coding region	Linear regression of neighbour-joining nucleotide substitution estimates against the years of isolation	(80)	139
Group V/(-)ssRNA virus	Paramyxoviridae	Metapneumovirus	Avian metapneumovirus	1.39E-3	9	F coding region	Tip-date calibration under the Bayesian phylogenetic framework	(81)	234
Group V/(-)ssRNA virus	Paramyxoviridae	Metapneumovirus	Avian metapneumovirus	6.14E-3	9	M2 coding region	Tip-date calibration under the Bayesian phylogenetic framework	(81)	258
Group V/(-)ssRNA virus	Paramyxoviridae	Metapneumovirus	Avian metapneumovirus	4.47E-3	9	N coding region	Tip-date calibration under the Bayesian phylogenetic framework	(81)	259
Group V/(-)ssRNA virus	Paramyxoviridae	Metapneumovirus	Avian metapneumovirus	7.01E-3	9	P coding region	Tip-date calibration under the Bayesian phylogenetic framework	(81)	249
Group V/(-)ssRNA virus	Paramyxoviridae	Metapneumovirus	Human metapneumovirus	4.58E-3	4	G coding region	Tip-date calibration under the Bayesian phylogenetic framework	(82)	149
Group V/(-)ssRNA virus	Paramyxoviridae	Metapneumovirus	Human metapneumovirus	7.87E-3	6	G coding region	Tip-date calibration under the Bayesian phylogenetic framework	(82)	156
Group V/(-)ssRNA virus	Paramyxoviridae	Metapneumovirus	Human metapneumovirus	6.14E-3	21	G coding region	Tip-date calibration under the Bayesian phylogenetic framework	(82)	127
Group V/(-)ssRNA virus	Paramyxoviridae	Metapneumovirus	Human metapneumovirus	6.48E-3	21	G coding region	Tip-date calibration under the Bayesian phylogenetic framework	(82)	137
Group V/(-)ssRNA virus	Paramyxoviridae	Metapneumovirus	Human metapneumovirus	5.18E-3	23	G coding region	Tip-date calibration under the Bayesian phylogenetic framework	(82)	145
Group V/(-)ssRNA virus	Paramyxoviridae	Metapneumovirus	Human metapneumovirus	5.34E-3	23	G coding region	Tip-date calibration under the Bayesian phylogenetic framework	(82)	140
Group V/(-)ssRNA virus	Paramyxoviridae	Metapneumovirus	Human metapneumovirus	7.12E-4	25	F coding region	Tip-date calibration under the Bayesian phylogenetic framework	(83)	146
Group V/(-)ssRNA virus	Paramyxoviridae	Morbillivirus	Canine distemper virus	1.17E-3	19	H coding region	Tip-date calibration under the Bayesian phylogenetic framework	(84)	1000
Group V/(-)ssRNA virus	Paramyxoviridae	Morbillivirus	Measles virus	6.58E-4	29	H coding region	Tip-date calibration under the Bayesian phylogenetic framework	(84)	186
Group V/(-)ssRNA virus	Paramyxoviridae	Morbillivirus	Measles virus	8.69E-4	26	N coding region	Tip-date calibration under the Bayesian phylogenetic framework	(84)	213

Group V/(-)ssRNA virus	Paramyxoviridae	Morbillivirus	Measles virus	3.4E-4	33	H coding region	Tip-date calibration under the maximum-likelihood phylogenetic framework	(85)	178
Group V/(-)ssRNA virus	Paramyxoviridae	Morbillivirus	Measles virus	6.44E-4	55	H coding region	Tip-date calibration under the Bayesian phylogenetic framework	(86)	201
Group V/(-)ssRNA virus	Paramyxoviridae	Morbillivirus	Measles virus	6.02E-4	55	N coding region	Tip-date calibration under the Bayesian phylogenetic framework	(86)	222
Group V/(-)ssRNA virus	Paramyxoviridae	Pneumovirus	Human respiratory syncytial virus	1.83E-3	47	G coding region	Tip-date calibration under the maximum-likelihood phylogenetic framework	(87)	349
Group V/(-)ssRNA virus	Paramyxoviridae	Pneumovirus	Human respiratory syncytial virus	1.95E-3	44	G coding region	Tip-date calibration under the maximum-likelihood phylogenetic framework	(88)	329
Group V/(-)ssRNA virus	Paramyxoviridae	Pneumovirus	Human respiratory syncytial virus	2.5E-3	5	G coding region	Tip-date calibration under the maximum-likelihood phylogenetic framework	(89)	322
Group V/(-)ssRNA virus	Paramyxoviridae	Pneumovirus	Respiratory syncytial virus	2.31E-3	10	G coding region	Tip-date calibration under the Bayesian phylogenetic framework	(90)	1000
Group V/(-)ssRNA virus	Paramyxoviridae	Rubulavirus	Mumps virus	9.17E-4	54	HN coding region	Tip-date calibration under the Bayesian phylogenetic framework	(84)	1000
Group V/(-)ssRNA virus	Rhabdoviridae	Lyssavirus	Rabies virus	5.10E-5	35	G coding region	Tip-date calibration under the Bayesian phylogenetic framework	(91)	76
Group V/(-)ssRNA virus	Rhabdoviridae	Lyssavirus	Rabies virus	6.11E-5	35	N coding region	Tip-date calibration under the Bayesian phylogenetic framework	(91)	48
Group V/(-)ssRNA virus	Rhabdoviridae	Lyssavirus	Rabies virus	2.32E-4	27	N coding region	Tip-date calibration under the Bayesian phylogenetic framework	(92)	54
Group V/(-)ssRNA virus	Rhabdoviridae	Lyssavirus	Rabies virus	1.53E-4	40	G coding region	Tip-date calibration under the Bayesian phylogenetic framework	(93)	66
Group V/(-)ssRNA virus	Rhabdoviridae	Lyssavirus	Rabies virus	1.68E-3	13	G-L intergenic region	Tip-date calibration under the Bayesian phylogenetic framework	(94)	62
Group V/(-)ssRNA virus	Rhabdoviridae	Lyssavirus	Rabies virus	8.26E-4	20	G-L intergenic region	Tip-date calibration under the Bayesian phylogenetic framework	(94)	66
Group V/(-)ssRNA virus	Rhabdoviridae	Lyssavirus	Rabies virus	3.57E-4	14	N coding region	Tip-date calibration under the Bayesian phylogenetic framework	(95)	53
Group V/(-)ssRNA virus	Rhabdoviridae	Lyssavirus	Rabies virus	2.75E-4	16	N coding region	Tip-date calibration under the Bayesian phylogenetic framework	(95)	62
Group V/(-)ssRNA virus	Rhabdoviridae	Lyssavirus	Rabies virus	2.57E-4	19	N coding region	Tip-date calibration under the Bayesian phylogenetic framework	(95)	58
Group V/(-)ssRNA virus	Rhabdoviridae	Lyssavirus	Rabies virus	3.90E-4	20	N coding region	Tip-date calibration under the Bayesian phylogenetic framework	(95)	75
Group V/(-)ssRNA virus	Rhabdoviridae	Lyssavirus	Rabies virus	3.32E-4	26	N coding region	Tip-date calibration under the Bayesian phylogenetic framework	(95)	61
Group V/(-)ssRNA virus	Rhabdoviridae	Lyssavirus	Rabies virus	2.59E-4	20	P coding region	Tip-date calibration under the Bayesian phylogenetic framework	(95)	64
Group V/(-)ssRNA virus	Rhabdoviridae	Lyssavirus	Rabies virus	2.7E-4	33	N coding region	Tip-date calibration under the Bayesian phylogenetic framework	(96)	68
Group V/(-)ssRNA virus	Rhabdoviridae	Lyssavirus	Rabies virus	3.9E-4	25	G coding region	Tip-date calibration under the Bayesian phylogenetic framework	(97)	69
Group V/(-)ssRNA virus	Rhabdoviridae	Lyssavirus	Rabies virus	2.3E-4	36	N coding region	Tip-date calibration under the Bayesian phylogenetic framework	(97)	55

Group V/(-)ssRNA virus	Rhabdoviridae	Lyssavirus	Rabies virus	3.96E-4	39	G coding region	Tip-date calibration under the Bayesian phylogenetic framework	(98)	63
Group VI/RT-RNA virus	Retroviridae	Deltaretrovirus	Human T-lymphotropic virus 1	5.15E-6	760	Env coding region	Internal-node calibration under the maximum-likelihood phylogenetic framework	(99)	182
Group VI/RT-RNA virus	Retroviridae	Deltaretrovirus	Human T-lymphotropic virus 1	1.77E-6	760	LTR	Internal-node calibration under the maximum-likelihood phylogenetic framework	(99)	206
Group VI/RT-RNA virus	Retroviridae	Deltaretrovirus	Human T-lymphotropic virus 2	4.32E-5	62.5	LTR	Dividing an average maximum-likelihood nucleotide substitution estimate by the timescale of evolution	(100)	339
Group VI/RT-RNA virus	Retroviridae	Deltaretrovirus	Human T-lymphotropic virus 2	2.7E-4	14	LTR	Dividing the difference between neighbour–joining nucleotide substitution estimates by the difference between the years of isolation	(101, 102)	333
Group VI/RT-RNA virus	Retroviridae	Lentivirus	Feline immunodeficiency virus	3.03E-3	1	Env coding region	Internal-node calibration under the Bayesian phylogenetic framework	(103)	27
Group VI/RT-RNA virus	Retroviridae	Lentivirus	Feline immunodeficiency virus	2.48E-3	2	Env coding region	Internal-node calibration under the Bayesian phylogenetic framework	(103)	26
Group VI/RT-RNA virus	Retroviridae	Lentivirus	Feline immunodeficiency virus	3.89E-3	3	Env coding region	Internal-node calibration under the Bayesian phylogenetic framework	(103)	28
Group VI/RT-RNA virus	Retroviridae	Lentivirus	Feline immunodeficiency virus	3.47E-3	4	Env coding region	Internal-node calibration under the Bayesian phylogenetic framework	(103)	30
Group VI/RT-RNA virus	Retroviridae	Lentivirus	Feline immunodeficiency virus	3.00E-3	5	Env coding region	Internal-node calibration under the Bayesian phylogenetic framework	(103)	28
Group VI/RT-RNA virus	Retroviridae	Lentivirus	Feline immunodeficiency virus	1.08E-3	6	Env coding region	Internal-node calibration under the Bayesian phylogenetic framework	(103)	32
Group VI/RT-RNA virus	Retroviridae	Lentivirus	Feline immunodeficiency virus	1.27E-3	7	Env coding region	Internal-node calibration under the Bayesian phylogenetic framework	(103)	20
Group VI/RT-RNA virus	Retroviridae	Lentivirus	Feline immunodeficiency virus	1.19E-3	7	Env coding region	Internal-node calibration under the Bayesian phylogenetic framework	(103)	24
Group VI/RT-RNA virus	Retroviridae	Lentivirus	Feline immunodeficiency virus	1.67E-3	8	Env coding region	Internal-node calibration under the Bayesian phylogenetic framework	(103)	33
Group VI/RT-RNA virus	Retroviridae	Lentivirus	Feline immunodeficiency virus	1.32E-3 (1.14E-3, 1.54E-3)	4.1	Env coding region	Tip-date calibration under the Bayesian phylogenetic framework	(103)	23
Group VI/RT-RNA virus	Retroviridae	Lentivirus	Feline immunodeficiency virus	0.66E-3	0.7	Env coding region	Tip-date calibration under the maximum-likelihood phylogenetic framework	(103)	17
Group VI/RT-RNA virus	Retroviridae	Lentivirus	Feline immunodeficiency virus	1.54E-3	0.8	Env coding region	Tip-date calibration under the maximum-likelihood phylogenetic framework	(103)	28
Group VI/RT-RNA virus	Retroviridae	Lentivirus	Feline immunodeficiency virus	0.06E-3	1.0	Env coding region	Tip-date calibration under the maximum-likelihood phylogenetic framework	(103)	30
Group VI/RT-RNA virus	Retroviridae	Lentivirus	Feline immunodeficiency virus	2.57E-3	1.3	Env coding region	Tip-date calibration under the maximum-likelihood phylogenetic framework	(103)	32
Group VI/RT-RNA virus	Retroviridae	Lentivirus	Feline immunodeficiency virus	2.30E-3	1.6	Env coding region	Tip-date calibration under the maximum-likelihood phylogenetic framework	(103)	25

Group VI/RT-RNA virus	Retroviridae	Lentivirus	Feline immunodeficiency virus	3.88E-3	2.3	Env coding region	Tip-date calibration under the maximum-likelihood phylogenetic framework	(103)	23
Group VI/RT-RNA virus	Retroviridae	Lentivirus	Feline immunodeficiency virus	0.13E-3	2.5	Env coding region	Tip-date calibration under the maximum-likelihood phylogenetic framework	(103)	31
Group VI/RT-RNA virus	Retroviridae	Lentivirus	Feline immunodeficiency virus	0.65E-3	2.9	Env coding region	Tip-date calibration under the maximum-likelihood phylogenetic framework	(103)	24
Group VI/RT-RNA virus	Retroviridae	Lentivirus	Feline immunodeficiency virus	0.95E-3	4.1	Env coding region	Tip-date calibration under the maximum-likelihood phylogenetic framework	(103)	27
Group VI/RT-RNA virus	Retroviridae	Lentivirus	Feline immunodeficiency virus	4.75E-3	1	Pol coding region	Internal-node calibration under the Bayesian phylogenetic framework	(103)	33
Group VI/RT-RNA virus	Retroviridae	Lentivirus	Feline immunodeficiency virus	6.21E-3	2	Pol coding region	Internal-node calibration under the Bayesian phylogenetic framework	(103)	29
Group VI/RT-RNA virus	Retroviridae	Lentivirus	Feline immunodeficiency virus	5.87E-3	3	Pol coding region	Internal-node calibration under the Bayesian phylogenetic framework	(103)	19
Group VI/RT-RNA virus	Retroviridae	Lentivirus	Feline immunodeficiency virus	2.83E-3	4	Pol coding region	Internal-node calibration under the Bayesian phylogenetic framework	(103)	30
Group VI/RT-RNA virus	Retroviridae	Lentivirus	Feline immunodeficiency virus	1.85E-3	5	Pol coding region	Internal-node calibration under the Bayesian phylogenetic framework	(103)	31
Group VI/RT-RNA virus	Retroviridae	Lentivirus	Feline immunodeficiency virus	1.22E-3	6	Pol coding region	Internal-node calibration under the Bayesian phylogenetic framework	(103)	23
Group VI/RT-RNA virus	Retroviridae	Lentivirus	Feline immunodeficiency virus	1.07E-3	7	Pol coding region	Internal-node calibration under the Bayesian phylogenetic framework	(103)	23
Group VI/RT-RNA virus	Retroviridae	Lentivirus	Feline immunodeficiency virus	1.38E-3	7	Pol coding region	Internal-node calibration under the Bayesian phylogenetic framework	(103)	19
Group VI/RT-RNA virus	Retroviridae	Lentivirus	Feline immunodeficiency virus	1.03E-3	8	Pol coding region	Internal-node calibration under the Bayesian phylogenetic framework	(103)	33
Group VI/RT-RNA virus	Retroviridae	Lentivirus	Feline immunodeficiency virus	1.10E-3 (0.77E-3, 1.54E-3)	4.1	Pol coding region	Tip-date calibration under the Bayesian phylogenetic framework	(103)	20
Group VI/RT-RNA virus	Retroviridae	Lentivirus	Feline immunodeficiency virus	2.28E-3	0.7	Pol coding region	Tip-date calibration under the maximum-likelihood phylogenetic framework	(103)	21
Group VI/RT-RNA virus	Retroviridae	Lentivirus	Feline immunodeficiency virus	1.46E-3	0.8	Pol coding region	Tip-date calibration under the maximum-likelihood phylogenetic framework	(103)	27
Group VI/RT-RNA virus	Retroviridae	Lentivirus	Feline immunodeficiency virus	1.84E-3	1.0	Pol coding region	Tip-date calibration under the maximum-likelihood phylogenetic framework	(103)	28
Group VI/RT-RNA virus	Retroviridae	Lentivirus	Feline immunodeficiency virus	0.52E-3	1.3	Pol coding region	Tip-date calibration under the maximum-likelihood phylogenetic framework	(103)	24
Group VI/RT-RNA virus	Retroviridae	Lentivirus	Feline immunodeficiency virus	0.72E-3	1.6	Pol coding region	Tip-date calibration under the maximum-likelihood phylogenetic framework	(103)	29
Group VI/RT-RNA virus	Retroviridae	Lentivirus	Feline immunodeficiency virus	2.35E-3	2.3	Pol coding region	Tip-date calibration under the maximum-likelihood phylogenetic framework	(103)	27
Group VI/RT-RNA virus	Retroviridae	Lentivirus	Feline immunodeficiency virus	1.35E-3	2.5	Pol coding region	Tip-date calibration under the maximum-likelihood phylogenetic framework	(103)	23
Group VI/RT-RNA virus	Retroviridae	Lentivirus	Feline immunodeficiency virus	0.50E-3	2.9	Pol coding region	Tip-date calibration under the maximum-likelihood phylogenetic framework	(103)	27
Group VI/RT-RNA virus	Retroviridae	Lentivirus	Feline immunodeficiency virus	0.39E-3	4.1	Pol coding region	Tip-date calibration under the maximum-likelihood phylogenetic framework	(103)	26

Group VI/RT-RNA virus	Retroviridae	Lentivirus	Human immunodeficiency virus (nested in simian immunodeficiency virus)	0.96E-3	14	Pol coding region	Dividing an average neighbour-joining nucleotide substitution estimates by the timescale of evolution	(104)	21
Group VI/RT-RNA virus	Retroviridae	Lentivirus	Human immunodeficiency virus (nested in simian immunodeficiency virus)	1.08E-2	7	Vpu coding region	Tip-date calibration under the Bayesian phylogenetic framework	(105)	16
Group VI/RT-RNA virus	Retroviridae	Lentivirus	Human immunodeficiency virus (nested in simian immunodeficiency virus)	1.13E-3	2	Genome	Tip-date calibration under the Bayesian phylogenetic framework	(106)	11
Group VI/RT-RNA virus	Retroviridae	Lentivirus	Human immunodeficiency virus (nested in simian immunodeficiency virus)	1.42E-3	2	Genome	Tip-date calibration under the Bayesian phylogenetic framework	(106)	14
Group VI/RT-RNA virus	Retroviridae	Lentivirus	Human immunodeficiency virus (nested in simian immunodeficiency virus)	1.55E-3	2	Genome	Tip-date calibration under the Bayesian phylogenetic framework	(106)	14
Group VI/RT-RNA virus	Retroviridae	Lentivirus	Human immunodeficiency virus (nested in simian immunodeficiency virus)	1.55E-3	2	Genome	Tip-date calibration under the Bayesian phylogenetic framework	(106)	10
Group VI/RT-RNA virus	Retroviridae	Lentivirus	Human immunodeficiency virus (nested in simian immunodeficiency virus)	1.89E-3	2	Genome	Tip-date calibration under the Bayesian phylogenetic framework	(106)	16
Group VI/RT-RNA virus	Retroviridae	Lentivirus	Human immunodeficiency virus (nested in simian immunodeficiency virus)	2.41E-3	2	Genome	Tip-date calibration under the Bayesian phylogenetic framework	(106)	26
Group VI/RT-RNA virus	Retroviridae	Lentivirus	Human immunodeficiency virus (nested in simian immunodeficiency virus)	2.46E-3	2	Genome	Tip-date calibration under the Bayesian phylogenetic framework	(106)	14
Group VI/RT-RNA virus	Retroviridae	Lentivirus	Human immunodeficiency virus (nested in simian immunodeficiency virus)	2.55E-3	2	Genome	Tip-date calibration under the Bayesian phylogenetic framework	(106)	17
Group VI/RT-RNA virus	Retroviridae	Lentivirus	Human immunodeficiency virus (nested in simian immunodeficiency virus)	2.56E-3	2	Genome	Tip-date calibration under the Bayesian phylogenetic framework	(106)	19
Group VI/RT-RNA virus	Retroviridae	Lentivirus	Human immunodeficiency virus (nested in simian immunodeficiency virus)	3.63E-3	2	Genome	Tip-date calibration under the Bayesian phylogenetic framework	(106)	16
Group VI/RT-RNA virus	Retroviridae	Lentivirus	Human immunodeficiency virus (nested in simian immunodeficiency virus)	1.74E-3	2	Genome	Tip-date calibration under the Bayesian phylogenetic framework	(106)	18
Group VI/RT-RNA virus	Retroviridae	Lentivirus	Human immunodeficiency virus (nested in simian immunodeficiency virus)	1.41E-3	2	Genome	Tip-date calibration under the Bayesian phylogenetic framework	(106)	24
Group VI/RT-RNA virus	Retroviridae	Lentivirus	Human immunodeficiency virus (nested in simian immunodeficiency virus)	1.25E-3	2	Genome	Tip-date calibration under the Bayesian phylogenetic framework	(106)	26

Group VI/RT-RNA virus	Retroviridae	Lentivirus	Human immunodeficiency virus (nested in simian immunodeficiency virus)	1.35E-3	2	Genome	Tip-date calibration under the Bayesian phylogenetic framework	(106)	21
Group VI/RT-RNA virus	Retroviridae	Lentivirus	Human immunodeficiency virus (nested in simian immunodeficiency virus)	1.04E-3	2	Genome	Tip-date calibration under the Bayesian phylogenetic framework	(106)	19
Group VI/RT-RNA virus	Retroviridae	Lentivirus	Human immunodeficiency virus (nested in simian immunodeficiency virus)	2.06E-3	2	Genome	Tip-date calibration under the Bayesian phylogenetic framework	(106)	19
Group VI/RT-RNA virus	Retroviridae	Lentivirus	Human immunodeficiency virus (nested in simian immunodeficiency virus)	2.25E-3	2	Genome	Tip-date calibration under the Bayesian phylogenetic framework	(106)	17
Group VI/RT-RNA virus	Retroviridae	Lentivirus	Human immunodeficiency virus (nested in simian immunodeficiency virus)	3.43E-3	2	Genome	Tip-date calibration under the Bayesian phylogenetic framework	(106)	12
Group VI/RT-RNA virus	Retroviridae	Lentivirus	Human immunodeficiency virus (nested in simian immunodeficiency virus)	2.25E-3	2	Genome	Tip-date calibration under the Bayesian phylogenetic framework	(106)	13
Group VI/RT-RNA virus	Retroviridae	Lentivirus	Human immunodeficiency virus (nested in simian immunodeficiency virus)	2.39E-3	2	Genome	Tip-date calibration under the Bayesian phylogenetic framework	(106)	25
Group VI/RT-RNA virus	Retroviridae	Lentivirus	Human immunodeficiency virus (nested in simian immunodeficiency virus)	2.50E-3	11	Pol coding region	Tip-date calibration under the Bayesian phylogenetic framework	(107)	15
Group VI/RT-RNA virus	Retroviridae	Lentivirus	Human immunodeficiency virus (nested in simian immunodeficiency virus)	1.80E-3	12	Pol coding region	Tip-date calibration under the Bayesian phylogenetic framework	(107)	16
Group VI/RT-RNA virus	Retroviridae	Lentivirus	Human immunodeficiency virus (nested in simian immunodeficiency virus)	2.06E-3	15	Pol coding region	Tip-date calibration under the Bayesian phylogenetic framework	(108)	21
Group VI/RT-RNA virus	Retroviridae	Lentivirus	Human immunodeficiency virus (nested in simian immunodeficiency virus)	3.15E-3	20	Pol coding region	Tip-date calibration under the Bayesian phylogenetic framework	(109)	15
Group VI/RT-RNA virus	Retroviridae	Lentivirus	Human immunodeficiency virus (nested in simian immunodeficiency virus)	4.65E-3	14	Env coding region	Tip-date calibration under the Bayesian phylogenetic framework	(110)	15
Group VII/RT-DNA virus	Hepadnaviridae	Avihepadnavirus	Avian hepatitis B virus	7.32E-4	20	Genome	Tip-date calibration under the Bayesian phylogenetic framework	(111)	38
Group VII/RT-DNA virus	Hepadnaviridae	Orthohepadnavirus	Human hepatitis B virus	7.72E-4	22	Genome	Tip-date calibration under the Bayesian phylogenetic framework	(111)	93
Group VII/RT-DNA virus	Hepadnaviridae	Orthohepadnavirus	Human hepatitis B virus	3.39E-5 (2.60E-5, 4.43E-5)	21	Genome	Tip-date calibration under the Bayesian phylogenetic framework	(112)	92
Group VII/RT-DNA virus	Hepadnaviridae	Orthohepadnavirus	Human hepatitis B virus	6.15E-5 (6.10E-5, 6.20E-5)	23	Genome	Tip-date calibration under the Bayesian phylogenetic framework	(112)	102
Group VII/RT-DNA virus	Hepadnaviridae	Orthohepadnavirus	Human hepatitis B virus	8.1E-5	17	Genome	Tip-date calibration under the Bayesian phylogenetic framework	(113)	85

Group VII/RT-DNA virus	Hepadnaviridae	Orthohepadnavirus	Human hepatitis B virus	1.2E-5	17.5	Genome	Tip-date calibration under the Bayesian phylogenetic framework	(113)	107
Group VII/RT-DNA virus	Hepadnaviridae	Orthohepadnavirus	Human hepatitis B virus	1.2E-5	18	Genome	Tip-date calibration under the Bayesian phylogenetic framework	(113)	99
Group VII/RT-DNA virus	Hepadnaviridae	Orthohepadnavirus	Human hepatitis B virus	2.1E-4	18	Genome	Tip-date calibration under the Bayesian phylogenetic framework	(113)	103
Group VII/RT-DNA virus	Hepadnaviridae	Orthohepadnavirus	Human hepatitis B virus	3.0E-5	18.6	Genome	Tip-date calibration under the Bayesian phylogenetic framework	(113)	86
Group VII/RT-DNA virus	Hepadnaviridae	Orthohepadnavirus	Human hepatitis B virus	8.6E-5	18.9	Genome	Tip-date calibration under the Bayesian phylogenetic framework	(113)	100
Group VII/RT-DNA virus	Hepadnaviridae	Orthohepadnavirus	Human hepatitis B virus	8.0E-6	20.1	Genome	Tip-date calibration under the Bayesian phylogenetic framework	(113)	95
**Long-term rate estimate**									
Group I/dsDNA virus	Herpesviridae	Cytomegalovirus, Muromegalovirus, Varicellovirus	Alpha and beta herpesvirus	3E-9	112E6	UL2-UL5-UL15-UL19-UL27-UL28-UL29-UL30	Linear regression of maximum-likelihood nucleotide substitution estimates against viral divergence dates, inferred under the viral-host co-speciation assumption	(114)	345
Group I/dsDNA virus	Herpesviridae	Simplexvirus, Varicellovirus	Alpha herpesvirus (nested in alpha and beta herpesvirus)	2.7E-9	60E6	gB (excluding the 3rd codon position)	Linear regression of maximum-likelihood nucleotide substitution estimates against viral divergence dates, inferred under the viral-host co-speciation assumption	(115)	320
Group I/dsDNA virus	Herpesviridae	Simplexvirus	Human simplex virus 1 (nested in alpha herpesvirus, and alpha and beta herpesvirus)	1.82E-8	8.45E6	US7-US8	Internal-node calibration under the Bayesian phylogenetic framework	(116)	180
Group I/dsDNA virus	Papillomaviridae	Alphapapillomavirus, Betapapillomavirus, Gammapapillomavirus, Deltapapillomavirus, Epsilonpapillomavirus, Mupapillomavirus, Nupapillomavirus, Pipapillomavirus, Kappapapillomavirus, Sigmapapillomavirus, Xipapillomavirus, Dyoxipapillomavirus, Omikronpapillomavirus, Upsilonpapillomavirus, Taupapillomavirus, Lambdapapillomavirus	Papillomavirus	7.1E-9	95.65E6	E1 coding region	Internal-node calibration under the Bayesian phylogenetic framework	(117)	235
Group I/dsDNA virus	Papillomaviridae	Alphapapillomavirus, Betapapillomavirus, Gammapapillomavirus, Deltapapillomavirus, Epsilonpapillomavirus, Mupapillomavirus,	Papillomavirus	9.6E-9	95.65E6	L1 coding region	Internal-node calibration under the Bayesian phylogenetic framework	(117)	255

		Nupapillomavirus, Pipapillomavirus, Kappapapillomavirus, Sigmapapillomavirus, Xipapillomavirus, Dyoxipapillomavirus, Omikronpapillomavirus, Upsilonpapillomavirus, Taupapillomavirus, Lambdapapillomavirus							
Group I/dsDNA virus	Papillomaviridae	Dyozetapapillomavirus, Etapapillomavirus, Thetapapillomavirus	Avian and turtle papillomavirus (nested in papillomavirus)	1.1E-8	227.5E6	E1 coding region	Internal-node calibration under the Bayesian phylogenetic framework	(118)	136
Group I/dsDNA virus	Papillomaviridae	Dyozetapapillomavirus, Etapapillomavirus, Thetapapillomavirus	Avian and turtle papillomavirus (nested in papillomavirus)	1.2E-8	227.5E6	E2 coding region	Internal-node calibration under the Bayesian phylogenetic framework	(118)	123
Group I/dsDNA virus	Papillomaviridae	Dyozetapapillomavirus, Etapapillomavirus, Thetapapillomavirus	Avian and turtle papillomavirus (nested in papillomavirus)	0.9E-8	227.5E6	L1 coding region	Internal-node calibration under the Bayesian phylogenetic framework	(118)	135
Group I/dsDNA virus	Papillomaviridae	Dyozetapapillomavirus, Etapapillomavirus, Thetapapillomavirus	Avian and turtle papillomavirus (nested in papillomavirus)	2.2E-8	227.5E6	L2 coding region	Internal-node calibration under the Bayesian phylogenetic framework	(118)	116
Group I/dsDNA virus	Papillomaviridae	Lambdapapillomavirus	Feline papillomavirus (nested in papillomavirus)	1.95E-8	10.78E6	E6-E7-E1-E2-L2-L1	Internal-node calibration under the Bayesian phylogenetic framework	(119)	510
Group I/dsDNA virus	Polyomaviridae	Orthopolyomavirus	Human polyomavirus JC	2E-7	1E5	IG coding region	Dividing an average neighbour-joining nucleotide substitution estimate by the timescale of evolution, inferred under the viral-host co-speciation assumption	(120)	528
Group I/dsDNA virus	Poxviridae	Orthopoxvirus	Variola virus (Poxvirus)	5E-7	8600	35 conserved genes	Internal-node calibration under the maximum-likelihood phylogenetic framework	(121)	320
Group II/ssDNA virus	Geminiviridae	Begomovirus	South American begomovirus	5.79E-7	1.9E6	Coat protein coding region	Dividing an upper-bound maximum-likelihood nucleotide substitution estimate by the timescale of evolution, inferred under the viral-host co-speciation assumption	(122)	496
Group II/ssDNA virus	Geminiviridae	Mastrevirus	Wheat dwarf virus	1.33E-8	12E6	Genome	Dividing a neighbour-joining nucleotide substitution estimate by the timescale of evolution, inferred under the viral-host co-speciation assumption	(123)	1000
Group IV/(+)ssRNA virus	Virgaviridae	Tobamovirus	Tobamovirus	2.16E-8	100E6	Coat protein coding region	Internal-node calibration under the maximum-likelihood phylogenetic framework	(122)	1000
Group V/(-)ssRNA virus	Bunyaviridae	Hantavirus	Puumala and Tula hantavirus (nested in rodent hantavirus)	2.64E-7	4.75E6	S segment-N coding region	Dividing an average maximum-likelihood nucleotide substitution estimate by the timescale of evolution, inferred under the viral-host co-speciation assumption	(124)	489

Group V/(-)ssRNA virus	Bunyaviridae	Hantavirus	Puumala hantavirus (nested in Puumala and Tula hantavirus, and rodent hantavirus)	1.41E-7	2E6	S segment-N coding region	Dividing an average maximum-likelihood nucleotide substitution estimate by the timescale of evolution, inferred under the viral-host co-speciation assumption	(124)	252
Group V/(-)ssRNA virus	Bunyaviridae	Hantavirus	Rodent hantavirus	1.04E-6	10E6	S segment-N coding region	Dividing an average maximum-likelihood nucleotide substitution estimate by the timescale of evolution, inferred under the viral-host co-speciation assumption	(124)	259
Group VI/RT-RNA virus	Retroviridae	Deltaretrovirus	Human T-lymphotropic virus 1	1.54E-6	50000	LTR-Env	Internal-node calibration under the maximum-likelihood phylogenetic framework	(125)	205
Group VI/RT-RNA virus	Retroviridae	Deltaretrovirus	Human T-lymphotropic virus 1	2.1E-7	50000	Env coding region	Internal-node calibration under the Bayesian phylogenetic framework	(126)	190
Group VI/RT-RNA virus	Retroviridae	Deltaretrovirus	Human T-lymphotropic virus 1	5.6E-7	50000	LTR	Internal-node calibration under the Bayesian phylogenetic framework	(126)	217
Group VI/RT-RNA virus	Retroviridae	Deltaretrovirus	Human T-lymphotropic virus 2	1.02E-6	25000	LTR	Internal-node calibration under the maximum-likelihood phylogenetic framework	(101, 102)	328
Group VI/RT-RNA virus	Retroviridae	Lentivirus	Simian immunodeficiency virus	7.3E-6	10000	Pol coding region	Internal-node calibration under the Bayesian phylogenetic framework	(127)	530
Group VI/RT-RNA virus	Retroviridae	Spumavirus	Ape foamy virus (nested in Old World monkey and ape foamy virus, simian foamy virus, and foamy virus)	1.41E-8	16.52E6	Pol coding region	Dividing the Bayesian posterior distribution of nucleotide substitution estimate by the distribution of the timescale of evolution, inferred under the viral-host co-speciation assumption	(128)	54
Group VI/RT-RNA virus	Retroviridae	Spumavirus	Ape foamy virus (nested in Old World monkey and ape foamy virus, simian foamy virus, and foamy virus)	5.39E-8	2.17E6	Pol coding region	Dividing the Bayesian posterior distribution of nucleotide substitution estimate by the distribution of the timescale of evolution, inferred under the viral-host co-speciation assumption	(128)	53
Group VI/RT-RNA virus	Retroviridae	Spumavirus	Ape foamy virus (nested in Old World monkey and ape foamy virus, simian foamy virus, and foamy virus)	2.01E-8	8.30E6	Pol coding region	Dividing the Bayesian posterior distribution of nucleotide substitution estimate by the distribution of the timescale of evolution, inferred under the viral-host co-speciation assumption	(128)	52
Group VI/RT-RNA virus	Retroviridae	Spumavirus	Ape foamy virus (nested in Old World monkey and ape foamy virus, simian foamy virus, and foamy virus)	7.48E-8	9.6E5	Pol coding region	Dividing the Bayesian posterior distribution of nucleotide substitution estimate by the distribution of the timescale of evolution, inferred under the viral-host co-speciation assumption	(128)	66
Group VI/RT-RNA virus	Retroviridae	Spumavirus	Fereuungulata foamy virus (nested in foamy virus)	5.8E-9	88.7E6	Pol coding region	Dividing the Bayesian posterior distribution of nucleotide substitution estimate by the distribution of the timescale of evolution, inferred under the viral-host co-speciation assumption	(128)	750

Group VI/RT-RNA virus	Retroviridae	Spumavirus	Foamy virus	5.6E-9	98.9E6	Pol coding region	Dividing the Bayesian posterior distribution of nucleotide substitution estimate by the distribution of the timescale of evolution, inferred under the viral-host co-speciation assumption	(128)	250
Group VI/RT-RNA virus	Retroviridae	Spumavirus	Old World monkey and ape foamy virus (nested in simian foamy virus, and foamy virus)	1.70E-8	27.5E6	Pol coding region	Internal-node calibration under the maximum-likelihood phylogenetic framework	(129)	120
Group VI/RT-RNA virus	Retroviridae	Spumavirus	Old World monkey and ape foamy virus (nested in simian foamy virus, and foamy virus)	8.1E-9	31.56E6	Pol coding region	Dividing the Bayesian posterior distribution of nucleotide substitution estimate by the distribution of the timescale of evolution, inferred under the viral-host co-speciation assumption	(128)	123
Group VI/RT-RNA virus	Retroviridae	Spumavirus	Old World monkey foamy virus (nested in Old World monkey and ape foamy virus, simian foamy virus, and foamy virus)	1.27E-8	11.50E6	Pol coding region	Dividing the Bayesian posterior distribution of nucleotide substitution estimate by the distribution of the timescale of evolution, inferred under the viral-host co-speciation assumption	(128)	225
Group VI/RT-RNA virus	Retroviridae	Spumavirus	Simian foamy virus (nested in foamy virus)	7.79E-9	43E6	Pol coding region	Internal-node calibration under the Bayesian phylogenetic framework	(130)	145
Group VI/RT-RNA virus	Retroviridae	Spumavirus	Simian foamy virus (nested in foamy virus)	2.14E-8	43.47E6	Pol coding region	Internal-node calibration under the Bayesian phylogenetic framework	(131)	137
Group VII/RT-DNA virus	Hepadnaviridae	Avihepadnavirus	Avian hepatitis B virus	2.15E-8	19E6	Pol coding region	Dividing a maximum-likelihood nucleotide substitution estimate by the timescale of evolution, inferred under the viral-host co-speciation assumption	(132)	467
Group VII/RT-DNA virus	Hepadnaviridae	Avihepadnavirus	Avian hepatitis B virus	6.80E-8	19E6	Pol coding region	Dividing a maximum-likelihood nucleotide substitution estimate by the timescale of evolution, inferred under the viral-host co-speciation assumption	(132)	495
Group VII/RT-DNA virus	Hepadnaviridae	Orthohepadnavirus	Human hepatitis B virus	2.2E-6	6600	S/P region	Internal-node calibration under the Bayesian phylogenetic framework	(133)	38

†Values were directly taken from the original study.
When several estimates were available but the best-fit value was not indicated, a geometric mean was used.
When only the upper- and lower-bound rate estimates were reported, calculated based on the bound of evolutionary timescales, a harmonic mean rate estimate was used.
When only the upper- and lower-bound rate estimates were reported, calculated based on the bound of nucleotide substitution estimates, an arithmetic mean rate estimate was used.
*They were not sampled, and used in the TDRP analyses at the level of viral groups due to the sampling constraint criteria; however they were used in the TDRP analyses at the level of viral genera.

Reference

1. **Firth C**, **Kitchen A**, **Shapiro B**, **Suchard MA**, **Holmes EC**, **Rambaut A**. 2010. Using time-structured data to estimate evolutionary rates of double-stranded DNA viruses. Mol Biol Evol **27**:2038–2051.

2. **Shackelton LA**, **Rambaut A**, **Pybus OG**, **Holmes EC**. 2006. JC virus evolution and its association with human populations. J Virol **80**:9928–9933.

3. **Babkin I V.**, **Shchelkunov SN**. 2008. Molecular evolution of poxviruses. Russ J Genet **44**:895–908.

4. **Firth C**, **Charleston MA**, **Duffy S**, **Shapiro B**, **Holmes EC**. 2009. Insights into the evolutionary history of an emerging livestock pathogen: porcine circovirus 2. J Virol **83**:12813–12821.

5. **Duffy S**, **Holmes EC**. 2009. Validation of high rates of nucleotide substitution in geminiviruses: phylogenetic evidence from East African cassava mosaic viruses. J Gen Virol **90**:1539–1547.

6. **Lefeuvre P**, **Harkins GW**, **Lett J-M**, **Briddon RW**, **Chase MW**, **Moury B**, **Martin DP**. 2011. Evolutionary time-scale of the begomoviruses: evidence from integrated sequences in the Nicotiana genome. PLoS One **6**:e19193.

7. **Duffy S**, **Holmes EC**. 2008. Phylogenetic evidence for rapid rates of molecular evolution in the single-stranded DNA begomovirus tomato yellow leaf curl virus. J Virol **82**:957–965.

8. **Harkins GW**, **Delport W**, **Duffy S**, **Wood N**, **Monjane AL**, **Owor BE**, **Donaldson L**, **Saumtally S**, **Triton G**, **Briddon RW**, **Shepherd DN**, **Rybicki EP**, **Martin DP**, **Varsani A**. 2009. Experimental evidence indicating that mastreviruses probably did not co-diverge with their hosts. Virol J **6**:104.

9. **Almeida RPP**, **Bennett GM**, **Anhalt MD**, **Tsai CW**, **O'Grady P**. 2009. Spread of an introduced vector-borne banana virus in Hawaii. Mol Ecol **18**:136–146.

10. **Robles-Sikisaka R**, **Bohonak AJ**, **McClenaghan LR**, **Dhar AK**. 2010. Genetic signature of rapid IHHNV (infectious hypodermal and hematopoietic necrosis virus) expansion in wild Penaeus shrimp populations. PLoS One **5**:e11799.

11. **Shackelton LA**, **Holmes EC**. 2006. Phylogenetic evidence for the rapid evolution of human B19 erythrovirus. J Virol **80**:3666–3669.

12. **Parsyan A**, **Szmaragd C**, **Allain J-P**, **Candotti D**. 2007. Identification and genetic diversity of two human parvovirus B19 genotype 3 subtypes. J Gen Virol **88**:428–431.

13. **Norja P**, **Eis-Hübinger AM**, **Söderlund-Venermo M**, **Hedman K**, **Simmonds P**. 2008. Rapid sequence change and geographical spread of human parvovirus B19: comparison of B19 virus evolution in acute and persistent infections. J Virol **82**:6427–6433.

14. **Shackelton LA**, **Parrish CR**, **Truyen U**, **Holmes EC**. 2005. High rate of viral evolution associated with the emergence of carnivore parvovirus. Proc Natl Acad Sci U S A **102**:379–384.

15. **Streck AF**, **Bonatto SL**, **Homeier T**, **Souza CK**, **Gonçalves KR**, **Gava D**, **Canal CW**, **Truyen U**. 2011. High rate of viral evolution in the capsid protein of porcine parvovirus. J Gen Virol **92**:2628–2636.

16. **Song J**, **Shen D**, **Cui J**, **Zhao B**. 2010. Accelerated evolution of PRRSV during recent outbreaks in China. Virus Genes **41**:241–245.

17. **Forsberg R**. 2005. Divergence time of porcine reproductive and respiratory syndrome virus subtypes. Mol Biol Evol **22**:2131–2134.

18. **Hanada K**, **Suzuki Y**, **Gojobori T**. 2004. A large variation in the rates of synonymous substitution for RNA viruses and its relationship to a diversity of viral infection and transmission modes. Mol Biol Evol **21**:1074–1080.

19. **Kerr PJ**, **Kitchen A**, **Holmes EC**. 2009. Origin and phylodynamics of rabbit hemorrhagic disease virus. J Virol **83**:12129–12138.

20. **Bok K**, **Abente EJ**, **Realpe-Quintero M**, **Mitra T**, **Sosnovtsev S V**, **Kapikian AZ**, **Green KY**. 2009. Evolutionary dynamics of GII.4 noroviruses over a 34-year period. J Virol **83**:11890–11901.

21. **Boon D**, **Mahar JE**, **Abente EJ**, **Kirkwood CD**, **Purcell RH**, **Kapikian AZ**, **Green KY**, **Bok K**. 2011. Comparative Evolution of GII.3 and GII.4 Norovirus over a 31-Year Period. J Virol **85**:8656–8666.

22. **Pyrc K**, **Dijkman R**, **Deng L**, **Jebbink MF**, **Ross HA**, **Berkhout B**, **van der Hoek L**. 2006. Mosaic structure of human coronavirus NL63, one thousand years of evolution. J Mol Biol **364**:964–973.

23. **Vijgen L**, **Keyaerts E**, **Moës E**, **Thoelen I**, **Wollants E**, **Lemey P**, **Vandamme A-M**, **Van Ranst M**. 2005. Complete genomic sequence of human coronavirus OC43: molecular clock analysis suggests a relatively recent zoonotic coronavirus transmission event. J Virol **79**:1595–1604.

24. **Hon C-C**, **Lam T-Y**, **Shi Z-L**, **Drummond AJ**, **Yip C-W**, **Zeng F**, **Lam P-Y**, **Leung FC-C**. 2008. Evidence of the recombinant origin of a bat severe acute respiratory syndrome (SARS)-like coronavirus and its implications on the direct ancestor of SARS coronavirus. J Virol **82**:1819–1826.

25. **Lau SKP**, **Li KSM**, **Huang Y**, **Shek C-T**, **Tse H**, **Wang M**, **Choi GKY**, **Xu H**, **Lam CSF**, **Guo R**, **Chan K-H**, **Zheng B-J**, **Woo PCY**, **Yuen K-Y**. 2010. Ecoepidemiology and complete genome comparison of different strains of severe acute respiratory syndrome-related Rhinolophus bat coronavirus in China reveal bats as a reservoir for acute, self-limiting infection that allows recombination events. J Virol **84**:2808–2819.

26. **McKinley ET**, **Jackwood MW**, **Hilt DA**, **Kissinger JC**, **Robertson JS**, **Lemke C**, **Paterson AH**. 2011. Attenuated live vaccine usage affects accurate measures of virus diversity and mutation rates in avian coronavirus infectious bronchitis virus. Virus Res **158**:225–234.

27. **Twiddy SS**, **Holmes EC**, **Rambaut A**. 2003. Inferring the rate and time-scale of dengue virus evolution. Mol Biol Evol **20**:122–129.

28. **Patil JA**, **Cherian S**, **Walimbe AM**, **Patil BR**, **Sathe PS**, **Shah PS**, **Cecilia D**. 2011. Evolutionary dynamics of the

American African genotype of dengue type 1 virus in India (1962-2005). Infect Genet Evol **11**:1443–1448.

29. **Foster JE**, **Bennett SN**, **Carrington CVF**, **Vaughan H**, **McMillan WO**. 2004. Phylogeography and molecular evolution of dengue 2 in the Caribbean basin, 1981-2000. Virology **324**:48–59.

30. **Kumar SRP**, **Patil JA**, **Cecilia D**, **Cherian SS**, **Barde P V**, **Walimbe AM**, **Yadav PD**, **Yergolkar PN**, **Shah PS**, **Padbidri VS**, **Mishra AC**, **Mourya DT**. 2010. Evolution, dispersal and replacement of American genotype dengue type 2 viruses in India (1956-2005): selection pressure and molecular clock analyses. J Gen Virol **91**:707–720.

31. **Carrington CVF**, **Foster JE**, **Pybus OG**, **Bennett SN**, **Holmes EC**. 2005. Invasion and maintenance of dengue virus type 2 and type 4 in the Americas. J Virol **79**:14680–14687.

32. **Fajardo A**, **Recarey R**, **de Mora D**, **D'Andrea L**, **Alvarez M**, **Regato M**, **Colina R**, **Khan B**, **Cristina J**. 2009. Modeling gene sequence changes over time in type 3 dengue viruses from Ecuador. Virus Res **141**:105–109.

33. **Ramírez A**, **Fajardo A**, **Moros Z**, **Gerder M**, **Caraballo G**, **Camacho D**, **Comach G**, **Alarcón V**, **Zambrano J**, **Hernández R**, **Moratorio G**, **Cristina J**, **Liprandi F**. 2010. Evolution of dengue virus type 3 genotype III in Venezuela: diversification, rates and population dynamics. Virol J **7**:329.

34. **Araújo JMG**, **Nogueira RMR**, **Schatzmayr HG**, **Zanotto PM de A**, **Bello G**. 2009. Phylogeography and evolutionary history of dengue virus type 3. Infect Genet Evol **9**:716–725.

35. **Klungthong C**, **Zhang C**, **Mammen MP**, **Ubol S**, **Holmes EC**. 2004. The molecular epidemiology of dengue virus serotype 4 in Bangkok, Thailand. Virology **329**:168–179.

36. **Mohammed MAF**, **Galbraith SE**, **Radford AD**, **Dove W**, **Takasaki T**, **Kurane I**, **Solomon T**. 2011. Molecular phylogenetic and evolutionary analyses of Muar strain of Japanese encephalitis virus reveal it is the missing fifth genotype. Infect Genet Evol **11**:855–862.

37. **Mehla R**, **Kumar SRP**, **Yadav P**, **Barde P V**, **Yergolkar PN**, **Erickson BR**, **Carroll SA**, **Mishra AC**, **Nichol ST**, **Mourya DT**. 2009. Recent ancestry of Kyasanur Forest disease virus. Emerg Infect Dis **15**:1431–1437.

38. **Baillie GJ**, **Kolokotronis S-O**, **Waltari E**, **Maffei JG**, **Kramer LD**, **Perkins SL**. 2008. Phylogenetic and evolutionary analyses of St. Louis encephalitis virus genomes. Mol Phylogenet Evol **47**:717–728.

39. **Auguste AJ**, **Pybus OG**, **Carrington CVF**. 2009. Evolution and dispersal of St. Louis encephalitis virus in the Americas. Infect Genet Evol **9**:709–715.

40. **Bertolotti L**, **Kitron U**, **Goldberg TL**. 2007. Diversity and evolution of West Nile virus in Illinois and the United States, 2002-2005. Virology **360**:143–149.

41. **Bryant JE**, **Holmes EC**, **Barrett ADT**. 2007. Out of Africa: A molecular perspective on the introduction of yellow fever virus into the Americas. PLoS Pathog **3**:e75.

42. **Magiorkinis G**, **Magiorkinis E**, **Paraskevis D**, **Ho SYW**, **Shapiro B**, **Pybus OG**, **Allain JP**, **Hatzakis A**. 2009. The global spread of hepatitis C virus 1a and 1b: A phylodynamic and phylogeographic analysis. PLoS Med

6:e1000198.

43. **Gray RR**, **Parker J**, **Lemey P**, **Salemi M**, **Katzourakis A**, **Pybus OG**. 2011. The mode and tempo of hepatitis C virus evolution within and among hosts. BMC Evol Biol **11**:131.

44. **Romano CM**, **Zanotto PMDA**, **Holmes EC**. 2008. Bayesian coalescent analysis reveals a high rate of molecular evolution in GB virus C. J Mol Evol **66**:292–297.

45. **Pagán I**, **Holmes EC**. 2010. Long-term evolution of the Luteoviridae: Time scale and mode of virus speciation. J Virol **84**:6177–6187.

46. **Wu B**, **Blanchard-Letort A**, **Liu Y**, **Zhou G**, **Wang X**, **Elena SF**. 2011. Dynamics of molecular evolution and phylogeography of barley yellow dwarf virus-PAV. PLoS One **6**:e16896.

47. **Hicks AL**, **Duffy S**. 2011. Genus-specific substitution rate variability among picornaviruses. J Virol **85**:7942–7947.

48. **Yoon SH**, **Park W**, **King DP**, **Kim H**. 2011. Phylogenomics and molecular evolution of foot-and-mouth disease virus. Mol Cells **31**:413–421.

49. **Cottam EM**, **Haydon DT**, **Paton DJ**, **Gloster J**, **Wilesmith JW**, **Ferris NP**, **Hutchings GH**, **King DP**. 2006. Molecular epidemiology of the foot-and-mouth disease virus outbreak in the United Kingdom in 2001. J Virol **80**:11274–11282.

50. **Mirand A**, **Schuffenecker I**, **Henquell C**, **Billaud G**, **Jugie G**, **Falcon D**, **Mahul A**, **Archimbaud C**, **Terletskaia-Ladwig E**, **Diedrich S**, **Huemer HP**, **Enders M**, **Lina B**, **Peigue-Lafeuille H**, **Bailly J-L**. 2010. Phylogenetic evidence for a recent spread of two populations of human enterovirus 71 in European countries. J Gen Virol **91**:2263–2277.

51. **Chu PY**, **Lu PL**, **Tsai YL**, **Hsi E**, **Yao CY**, **Chen YH**, **Hsu LC**, **Wang SY**, **Wu HS**, **Lin YY**, **Su HJ**, **Lin KH**. 2011. Spatiotemporal phylogenetic analysis and molecular characterization of coxsackievirus A4. Infect Genet Evol **11**:1426–1435.

52. **Gullberg M**, **Tolf C**, **Jonsson N**, **Mulders MN**, **Savolainen-Kopra C**, **Hovi T**, **Van Ranst M**, **Lemey P**, **Hafenstein S**, **Lindberg AM**. 2010. Characterization of a putative ancestor of coxsackievirus B5. J Virol **84**:9695–9708.

53. **McWilliam Leitch EC**, **Bendig J**, **Cabrerizo M**, **Cardosa J**, **Hyypiä T**, **Ivanova OE**, **Kelly A**, **Kroes ACM**, **Lukashev A**, **MacAdam A**, **McMinn P**, **Roivainen M**, **Trallero G**, **Evans DJ**, **Simmonds P**. 2009. Transmission networks and population turnover of echovirus 30. J Virol **83**:2109–2118.

54. **Kew OM**, **Mulders MN**, **Lipskaya GY**, **da Silva EE**, **Patlansch MA**. 1995. Molecular epidemiology of polioviruses. Semin Virol **6**:401–414.

55. **Kulkarni MA**, **Walimbe AM**, **Cherian S**, **Arankalle VA**. 2009. Full length genomes of genotype IIIA Hepatitis A Virus strains (1995-2008) from India and estimates of the evolutionary rates and ages. Infect Genet Evol **9**:1287–1294.

56. **Moratorio G**, **Costa-Mattioli M**, **Piovani R**, **Romero H**, **Musto H**, **Cristina J**. 2007. Bayesian coalescent inference of hepatitis A virus populations: evolutionary rates and patterns. J Gen Virol **88**:3039–3042.

57. **Faria NR**, **de Vries M**, **van Hemert FJ**, **Benschop K**, **van der Hoek L**. 2009. Rooting human parechovirus evolution in time. BMC Evol Biol **9**:164.

58. **Gibbs AJ**, **Ohshima K**, **Phillips MJ**, **Gibbs MJ**. 2008. The prehistory of potyviruses: Their initial radiation was during the dawn of agriculture. PLoS One **3**:e2523.

59. **Simmons HE**, **Holmes EC**, **Stephenson AG**. 2008. Rapid evolutionary dynamics of zucchini yellow mosaic virus. J Gen Virol **89**:1081–1085.

60. **Padhi A**, **Moore AT**, **Brown MB**, **Foster JE**, **Pfeffer M**, **Gaines KP**, **O'Brien VA**, **Strickler SA**, **Johnson AE**, **Brown CR**. 2008. Phylogeographical structure and evolutionary history of two Buggy Creek virus lineages in the western Great Plains of North America. J Gen Virol **89**:2122–2131.

61. **Jones A**, **Lowry K**, **Aaskov J**, **Holmes EC**, **Kitchen A**. 2010. Molecular evolutionary dynamics of Ross River virus and implications for vaccine efficacy. J Gen Virol **91**:182–188.

62. **Auguste AJ**, **Volk SM**, **Arrigo NC**, **Martinez R**, **Ramkissoon V**, **Adams AP**, **Thompson NN**, **Adesiyun AA**, **Chadee DD**, **Foster JE**, **Travassos Da Rosa APA**, **Tesh RB**, **Weaver SC**, **Carrington CVF**. 2009. Isolation and phylogenetic analysis of Mucambo virus (Venezuelan equine encephalitis complex subtype IIIA) in Trinidad. Virology **392**:123–130.

63. **Fargette D**, **Pinel-Galzi A**, **Sérémé D**, **Lacombe S**, **Hébrard E**, **Traoré O**, **Konaté G**. 2008. Diversification of rice yellow mottle virus and related viruses spans the history of agriculture from the neolithic to the present. PLoS Pathog **4**:e1000125.

64. **Fargette D**, **Pinel A**, **Rakotomalala M**, **Sangu E**, **Traoré O**, **Sérémé D**, **Sorho F**, **Issaka S**, **Hébrard E**, **Séré Y**, **Kanyeka Z**, **Konaté G**. 2008. Rice yellow mottle virus, an RNA plant virus, evolves as rapidly as most RNA animal viruses. J Virol **82**:3584–3589.

65. **Pagán I**, **Firth C**, **Holmes EC**. 2010. Phylogenetic analysis reveals rapid evolutionary dynamics in the plant RNA virus genus tobamovirus. J Mol Evol **71**:298–307.

66. **Ramsden C**, **Melo FL**, **Figueiredo LM**, **Holmes EC**, **Zanotto PMA**, **Moreli ML**, **Moro De Sousa RL**, **Borges AA**, **Garcia De Figueiredo G**, **Bisordi I**, **Nagasse-Sugahara TK**, **Suzuki A**, **Pereira LE**, **Pereira De Souza R**, **Madia De Souza LT**, **Torres Braconi C**, **Araujo J**. 2008. High rates of molecular evolution in hantaviruses. Mol Biol Evol **25**:1488–1492.

67. **Ramsden C**, **Holmes EC**, **Charleston MA**. 2009. Hantavirus evolution in relation to its rodent and insectivore hosts: No evidence for codivergence. Mol Biol Evol **26**:143–153.

68. **Black WC**, **Doty JB**, **Hughes MT**, **Beaty BJ**, **Calisher CH**. 2009. Temporal and geographic evidence for evolution of Sin Nombre virus using molecular analyses of viral RNA from Colorado, New Mexico and Montana. Virol J **6**:102.

69. **Anagnostou V**, **Papa A**. 2009. Evolution of Crimean-Congo hemorrhagic fever virus. Infect Genet Evol **9**:948–954.

70. **Carroll SA**, **Bird BH**, **Rollin PE**, **Nichol ST**. 2010. Ancient common ancestry of Crimean-Congo hemorrhagic fever virus. Mol Phylogenet Evol **55**:1103–1110.

71. **Bird BH**, **Khristova ML**, **Rollin PE**, **Ksiazek TG**, **Nichol ST**. 2007. Complete genome analysis of 33 ecologically and biologically diverse Rift Valley fever virus strains reveals widespread virus movement and low genetic diversity due to recent common ancestry. J Virol **81**:2805–2816.

72. **Bird BH**, **Githinji JWK**, **Macharia JM**, **Kasiiti JL**, **Muriithi RM**, **Gacheru SG**, **Musaa JO**, **Towner JS**, **Reeder SA**, **Oliver JB**, **Stevens TL**, **Erickson BR**, **Morgan LT**, **Khristova ML**, **Hartman AL**, **Comer JA**, **Rollin PE**, **Ksiazek TG**, **Nichol ST**. 2008. Multiple virus lineages sharing recent common ancestry were associated with a Large Rift Valley fever outbreak among livestock in Kenya during 2006-2007. J Virol **82**:11152–11166.

73. **Smith GJD**, **Vijaykrishna D**, **Bahl J**, **Lycett SJ**, **Worobey M**, **Pybus OG**, **Ma SK**, **Cheung CL**, **Raghwani J**, **Bhatt S**, **Peiris JSM**, **Guan Y**, **Rambaut A**. 2009. Origins and evolutionary genomics of the 2009 swine-origin H1N1 influenza A epidemic. Nature **459**:1122–1125.

74. **Chen R**, **Holmes EC**. 2006. Avian influenza virus exhibits rapid evolutionary dynamics. Mol Biol Evol **23**:2336–2341.

75. **Lindstrom S**, **Endo A**, **Sugita S**, **Pecoraro M**, **Hiromoto Y**, **Kamada M**, **Takahashi T**, **Nerome K**. 1998. Phylogenetic analyses of the matrix and non-structural genes of equine influenza viruses. Arch Virol **143**:1585–1598.

76. **Goñi N**, **Fajardo A**, **Moratorio G**, **Colina R**, **Cristina J**. 2009. Modeling gene sequences over time in 2009 H1N1 influenza A virus populations. Virol J **6**:215.

77. **Fourment M**, **Wood JT**, **Gibbs AJ**, **Gibbs MJ**. 2010. Evolutionary dynamics of the N1 neuraminidases of the main lineages of influenza A viruses. Mol Phylogenet Evol **56**:526–535.

78. **Xu J**, **Christman MC**, **Donis RO**, **Lu G**. 2011. Evolutionary dynamics of influenza A nucleoprotein (NP) lineages revealed by large-scale sequence analyses. Infect Genet Evol **11**:2125–2132.

79. **Xu X**, **Cox NJ**, **Bender CA**, **Regnery HL**, **Shaw MW**. 1996. Genetic variation in neuraminidase genes of influenza A (H3N2) viruses. Virology **224**:175–183.

80. **Lindstrom SE**, **Hiromoto Y**, **Nishimura H**, **Saito T**, **Nerome R**, **Nerome K**. 1999. Comparative analysis of evolutionary mechanisms of the hemagglutinin and three internal protein genes of influenza B virus: multiple cocirculating lineages and frequent reassortment of the NP, M, and NS genes. J Virol **73**:4413–4426.

81. **Padhi A**, **Poss M**. 2009. Population dynamics and rates of molecular evolution of a recently emerged paramyxovirus, avian metapneumovirus subtype C. J Virol **83**:2015–2019.

82. **Padhi A**, **Verghese B**. 2008. Positive natural selection in the evolution of human metapneumovirus attachment glycoprotein. Virus Res **131**:121–131.

83. **Yang C-F**, **Wang CK**, **Tollefson SJ**, **Piyaratna R**, **Lintao LD**, **Chu M**, **Liem A**, **Mark M**, **Spaete RR**, **Crowe JE**, **Williams J V**. 2009. Genetic diversity and evolution of human metapneumovirus fusion protein over twenty years. Virol J **6**:138.

84. **Pomeroy LW**, **Bjørnstad ON**, **Holmes EC**. 2008. The evolutionary and epidemiological dynamics of the paramyxoviridae. J Mol Evol **66**:98–106.

85. **Woelk CH**, **Pybus OG**, **Jin L**, **Brown DWG**, **Holmes EC**. 2002. Increased positive selection pressure in persistent (SSPE) versus acute measles virus infections. J Gen Virol **83**:1419–1430.

86. **Furuse Y**, **Suzuki A**, **Oshitani H**. 2010. Origin of measles virus: divergence from rinderpest virus between the 11th and 12th centuries. Virol J **7**:52.

87. **Zlateva KT**, **Lemey P**, **Vandamme A-M**, **Van Ranst M**. 2004. Molecular evolution and circulation patterns of human respiratory syncytial virus subgroup A: Positively selected sites in the attachment G glycoprotein. J Virol **78**:4675–4683.

88. **Zlateva KT**, **Lemey P**, **Moës E**, **Vandamme A-M**, **Van Ranst M**. 2005. Genetic variability and molecular evolution of the human respiratory syncytial virus subgroup B attachment G protein. J Virol **79**:9157–9167.

89. **Trento A**, **Viegas M**, **Galiano M**, **Videla C**, **Carballal G**, **Mistchenko AS**, **Melero JA**. 2006. Natural history of human respiratory syncytial virus inferred from phylogenetic analysis of the attachment (G) glycoprotein with a 60-nucleotide duplication. J Virol **80**:975–984.

90. **van Niekerk S**, **Venter M**. 2011. Replacement of previously circulating respiratory syncytial virus subtype B strains with the BA genotype in South Africa. J Virol **85**:8789–8797.

91. **Davis PL**, **Holmes EC**, **Larrous F**, **Van der Poel WHM**, **Tjørnehøj K**, **Alonso WJ**, **Bourhy H**. 2005. Phylogeography, population dynamics, and molecular evolution of European bat lyssaviruses. J Virol **79**:10487–10497.

92. **Hughes GJ**, **Orciari LA**, **Rupprecht CE**. 2005. Evolutionary timescale of rabies virus adaptation to North American bats inferred from the substitution rate of the nucleoprotein gene. J Gen Virol **86**:1467–1474.

93. **Meng S**, **Xu G**, **Wu X**, **Lei Y**, **Yan J**, **Nadin-Davis SA**, **Liu H**, **Wu J**, **Wang D**, **Dong G**, **Yang X**, **Rupprecht CE**. 2010. Transmission dynamics of rabies in China over the last 40 years: 1969-2009. J Clin Virol **49**:47–52.

94. **Davis PL**, **Rambaut A**, **Bourhy H**, **Holmes EC**. 2007. The evolutionary dynamics of canid and mongoose rabies virus in Southern Africa. Arch Virol **152**:1251–1258.

95. **Davis PL**, **Bourhy H**, **Holmes EC**. 2006. The evolutionary history and dynamics of bat rabies virus. Infect Genet Evol **6**:464–473.

96. **David D**, **Hughes GJ**, **Yakobson BA**, **Davidson I**, **Un H**, **Aylan O**, **Kuzmin I V**, **Rupprecht CE**. 2007. Identification of novel canine rabies virus clades in the Middle East and North Africa. J Gen Virol **88**:967–980.

97. **Bourhy H**, **Reynes J-M**, **Dunham EJ**, **Dacheux L**, **Larrous F**, **Huong VTQ**, **Xu G**, **Yan J**, **Miranda MEG**, **Holmes EC**. 2008. The origin and phylogeography of dog rabies virus. J Gen Virol **89**:2673–2681.

98. **Ming P**, **Yan J**, **Rayner S**, **Meng S**, **Xu G**, **Tang Q**, **Wu J**, **Luo J**, **Yang X**. 2010. A history estimate and evolutionary analysis of rabies virus variants in China. J Gen Virol **91**:759–764.

99. **Van Dooren S**, **Pybus OG**, **Salemi M**, **Liu H-F**, **Goubau P**, **Remondegui C**, **Talarmin A**, **Gotuzzo E**, **Alcantara LCJ**, **Galvão-Castro B**, **Vandamme A-M**. 2004. The low evolutionary rate of human T-cell lymphotropic virus type-1 confirmed by analysis of vertical transmission chains. Mol Biol Evol **21**:603–611.

100. **Salemi M**, **Vandamme AM**, **Gradozzi C**, **Van Laethem K**, **Cattaneo E**, **Taylor G**, **Casoli C**, **Goubau P**, **Desmyter J**, **Bertazzoni U**. 1998. Evolutionary rate and genetic heterogeneity of human T-cell lymphotropic virus type II (HTLV-II) using isolates from European injecting drug users. J Mol Evol **46**:602–611.

101. **Salemi M**, **Lewis M**, **Egan JF**, **Hall WW**, **Desmyter J**, **Vandamme AM**. 1999. Different population dynamics of human T cell lymphotropic virus type II in intravenous drug users compared with endemically infected tribes. Proc Natl Acad Sci U S A **96**:13253–13258.

102. **Vandamme AM**, **Bertazzoni U**, **Salemi M**. 2000. Evolutionary strategies of human T-cell lymphotropic virus type II. Gene **261**:171–180.

103. **Biek R**, **Rodrigo AG**, **Holley D**, **Drummond A**, **Anderson CR**, **Ross HA**, **Poss M**. 2003. Epidemiology, genetic diversity, and evolution of endemic feline immunodeficiency virus in a population of wild cougars. J Virol **77**:9578–9589.

104. **Sharp PM**, **Li WH**. 1988. Understanding the origins of AIDS viruses. Nature **336**:315.

105. **Aulicino PC**, **Holmes EC**, **Rocco C**, **Mangano A**, **Sen L**. 2007. Extremely rapid spread of human immunodeficiency virus type 1 BF recombinants in Argentina. J Virol **81**:427–429.

106. **Lemey P**, **Van Dooren S**, **Vandamme A-M**. 2005. Evolutionary dynamics of human retroviruses investigated through full-genome scanning. Mol Biol Evol **22**:942–951.

107. **Bello G**, **Aulicino PC**, **Ruchansky D**, **Guimarães ML**, **Lopez-Galindez C**, **Casado C**, **Chiparelli H**, **Rocco C**, **Mangano A**, **Sen L**, **Morgado MG**. 2010. Phylodynamics of HIV-1 circulating recombinant forms 12_BF and 38_BF in Argentina and Uruguay. Retrovirology **7**:22.

108. **Dalai SC**, **de Oliveira T**, **Harkins GW**, **Kassaye SG**, **Lint J**, **Manasa J**, **Johnston E**, **Katzenstein D**. 2009. Evolution and molecular epidemiology of subtype C HIV-1 in Zimbabwe. AIDS **23**:2523–2532.

109. **Mehta SR**, **Wertheim JO**, **Delport W**, **Ene L**, **Tardei G**, **Duiculescu D**, **Pond SLK**, **Smith DM**. 2011. Using phylogeography to characterize the origins of the HIV-1 subtype F epidemic in Romania. Infect Genet Evol **11**:975–979.

110. **Lemey P**, **Rambaut A**, **Pybus OG**. 2006. HIV evolutionary dynamics within and among hosts. AIDS Rev **8**:125–140.

111. **Zhou Y**, **Holmes EC**. 2007. Bayesian estimates of the evolutionary rate and age of hepatitis B virus. J Mol Evol **65**:197–205.

112. **Harrison A**, **Lemey P**, **Hurles M**, **Moyes C**, **Horn S**, **Pryor J**, **Malani J**, **Supuri M**, **Masta A**, **Teriboriki B**, **Toatu T**, **Penny D**, **Rambaut A**, **Shapiro B**. 2011. Genomic analysis of hepatitis B virus reveals antigen state and genotype as sources of evolutionary rate variation. Viruses **3**:83–101.

113. **Wang H-Y**, **Chien M-H**, **Huang H-P**, **Chang H-C**, **Wu C-C**, **Chen P-J**, **Chang M-H**, **Chen D-S**. 2010. Distinct hepatitis B virus dynamics in the immunotolerant and early immunoclearance phases. J Virol **84**:3454–3463.

114. **McGeoch DJ**, **Dolan A**, **Ralph AC**. 2000. Toward a comprehensive phylogeny for mammalian and avian herpesviruses. J Virol **74**:10401–10406.

115. **McGeoch DJ**, **Cook S**. 1994. Molecular phylogeny of the alphaherpesvirinae subfamily and a proposed evolutionary timescale. J Mol Biol **238**:9–22.

116. **Norberg P**, **Tyler S**, **Severini A**, **Whitley R**, **Liljeqvist J-Å**, **Bergström T**. 2011. A genome-wide comparative evolutionary analysis of herpes simplex virus type 1 and varicella zoster virus. PLoS One **6**:e22527.

117. **Shah SD**, **Doorbar J**, **Goldstein RA**. 2010. Analysis of host-parasite incongruence in papillomavirus evolution using importance sampling. Mol Biol Evol **27**:1301–1314.

118. **Herbst LH**, **Lenz J**, **Van Doorslaer K**, **Chen Z**, **Stacy BA**, **Wellehan JFX**, **Manire CA**, **Burk RD**. 2009. Genomic characterization of two novel reptilian papillomaviruses, Chelonia mydas papillomavirus 1 and Caretta caretta papillomavirus 1. Virology **383**:131–135.

119. **Rector A**, **Lemey P**, **Tachezy R**, **Mostmans S**, **Ghim S-J**, **Van Doorslaer K**, **Roelke M**, **Bush M**, **Montali RJ**, **Joslin J**, **Burk RD**, **Jenson AB**, **Sundberg JP**, **Shapiro B**, **Van Ranst M**. 2007. Ancient papillomavirus-host co-speciation in Felidae. Genome Biol **8**:R57.

120. **Sugimoto C**, **Kitamura T**, **Guo J**, **Al-Ahdal MN**, **Shchelkunov SN**, **Otova B**, **Ondrejka P**, **Chollet JY**, **El-Safi S**, **Ettayebi M**, **Grésenguet G**, **Kocagöz T**, **Chaiyarasamee S**, **Thant KZ**, **Thein S**, **Moe K**, **Kobayashi N**, **Taguchi F**, **Yogo Y**. 1997. Typing of urinary JC virus DNA offers a novel means of tracing human migrations. Proc Natl Acad Sci U S A **94**:9191–9196.

121. **Babkin I V**, **Babkina IN**. 2011. Molecular dating in the evolution of vertebrate poxviruses. Intervirology **54**:253–260.

122. **Gibbs AJ**, **Fargette D**, **García-Arenal F**, **Gibbs MJ**. 2010. Time--the emerging dimension of plant virus studies. J Gen Virol **91**:13–22.

123. **Wu B**, **Melcher U**, **Guo X**, **Wang X**, **Fan L**, **Zhou G**. 2008. Assessment of codivergence of mastreviruses with their plant hosts. BMC Evol Biol **8**:335.

124. **Sironen T**, **Vaheri A**, **Plyusnin A**. 2001. Molecular evolution of Puumala hantavirus. J Virol **75**:11803–11810.

125. **Van Dooren S**, **Salemi M**, **Vandamme A-M**. 2001. Dating the origin of the African human T-Cell lymphotropic

virus type-I (HTLV-I) subtypes. Mol Biol Evol **18**:661–671.

126. **Lemey P**, **Pybus OG**, **Van Dooren S**, **Vandamme A-M**. 2005. A Bayesian statistical analysis of human T-cell lymphotropic virus evolutionary rates. Infect Genet Evol **5**:291–298.

127. **Worobey M**, **Telfer P**, **Souquière S**, **Hunter M**, **Coleman CA**, **Metzger MJ**, **Reed P**, **Makuwa M**, **Hearn G**, **Honarvar S**, **Roques P**, **Apetrei C**, **Kazanji M**, **Marx PA**. 2010. Island biogeography reveals the deep history of SIV. Science **329**:1487.

128. **Aiewsakun P**, **Katzourakis A**. 2015. Time dependency of foamy virus evolutionary rate estimates. BMC Evol Biol **15**:119.

129. **Switzer WM**, **Salemi M**, **Shanmugam V**, **Gao F**, **Cong M-E**, **Kuiken C**, **Bhullar V**, **Beer BE**, **Vallet D**, **Gautier-Hion A**, **Tooze Z**, **Villinger F**, **Holmes EC**, **Heneine W**. 2005. Ancient co-speciation of simian foamy viruses and primates. Nature **434**:376–380.

130. **Muniz CP**, **Troncoso LL**, **Moreira M a**, **Soares E a**, **Pissinatti A**, **Bonvicino CR**, **Seuánez HN**, **Sharma B**, **Jia H**, **Shankar A**, **Switzer WM**, **Santos AF**, **Soares M a**. 2013. Identification and characterization of highly divergent simian foamy viruses in a wide range of new world primates from Brazil. PLoS One **8**:e67568.

131. **Ghersi BM**, **Jia H**, **Aiewsakun P**, **Katzourakis A**, **Mendoza P**, **Bausch DG**, **Kasper MR**, **Montgomery JM**, **Switzer WM**. 2015. Wide distribution and ancient evolutionary history of simian foamy viruses in New World primates. Retrovirology **12**:89.

132. **Gilbert C**, **Feschotte C**. 2010. Genomic fossils calibrate the long-term evolution of hepadnaviruses. PLoS Biol **8**:e1000495.

133. **Paraskevis D**, **Magiorkinis G**, **Magiorkinis E**, **Ho SYW**, **Belshaw R**, **Allain J-P**, **Hatzakis A**. 2013. Dating the origin and dispersal of hepatitis B virus infection in humans and primates. Hepatology **57**:908–916.
